# Muscle transcriptomic profiles in pigs with divergent phenotypes for fatness traits

**DOI:** 10.1186/1471-2164-11-372

**Published:** 2010-06-11

**Authors:** Angela Cánovas, Raquel Quintanilla, Marcel Amills, Ramona N Pena

**Affiliations:** 1 IRTA, Genètica i Millora Animal, 191 Av Alcalde Rovira Roure, 25198 Lleida, Spain; 2 Departament de Ciència Animal i dels Aliments, Facultat de Veterinària, Universitat Autònoma de Barcelona, 08193 Bellaterra, Spain

## Abstract

**Background:**

Selection for increasing intramuscular fat content would definitively improve the palatability and juiciness of pig meat as well as the sensorial and organoleptic properties of cured products. However, evidences obtained in human and model organisms suggest that high levels of intramuscular fat might alter muscle lipid and carbohydrate metabolism. We have analysed this issue by determining the transcriptomic profiles of Duroc pigs with divergent phenotypes for 13 fatness traits. The strong aptitude of Duroc pigs to have high levels of intramuscular fat makes them a valuable model to analyse the mechanisms that regulate muscle lipid metabolism, an issue with evident implications in the elucidation of the genetic basis of human metabolic diseases such as obesity and insulin resistance.

**Results:**

Muscle gene expression profiles of 68 Duroc pigs belonging to two groups (HIGH and LOW) with extreme phenotypes for lipid deposition and composition traits have been analysed. Microarray and quantitative PCR analysis showed that genes related to fatty acid uptake, lipogenesis and triacylglycerol synthesis were upregulated in the muscle tissue of HIGH pigs, which are fatter and have higher amounts of intramuscular fat than their LOW counterparts. Paradoxically, lipolytic genes also showed increased mRNA levels in the HIGH group suggesting the existence of a cycle where triacylglycerols are continuously synthesized and degraded. Several genes related to the insulin-signalling pathway, that is usually impaired in obese humans, were also upregulated. Finally, genes related to antigen-processing and presentation were downregulated in the HIGH group.

**Conclusion:**

Our data suggest that selection for increasing intramuscular fat content in pigs would lead to a shift but not a disruption of the metabolic homeostasis of muscle cells. Future studies on the post-translational changes affecting protein activity or expression as well as information about protein location within the cell would be needed to to elucidate the effects of lipid deposition on muscle metabolism in pigs.

## Background

Muscle lipid metabolism affects a wide diversity of meat quality traits that are of huge importance for the pig industry [1]. Intramuscular fat percentage is favourably associated with meat texture, tenderness, flavour and juiciness [2,3]. In addition, muscle fat composition has a strong effect on the sensorial, nutritional and technological properties of meat [1]. The proportion of monounsaturated fatty acids (mainly oleic) is a key factor determining meat fat consistency and taste [1], whereas polyunsaturated fatty acids have a marked tendency to be oxidized, producing a rancid odour and taste that decrease meat consumer's acceptance. From a human health perspective, increased amounts of polyunsaturated fatty acids in the diet diminish the susceptibility to suffer cardiovascular diseases, while saturated fatty acids have the opposite effect [4].

Selection for higher muscle fat content and composition might have a long-term impact on pig muscle physiology that has not been evaluated yet. For instance, in obese humans, accumulation of triacylglycerols in the myocyte is associated with the development of insulin resistance, metabolic syndrome and type II diabetes [5,6]. In this way, increases in skeletal muscle fat stores are often accompanied by a parallel reduction in the β-oxidation of fatty acids and the progressive accumulation of lipid metabolites, such as diacylglycerol and long-chain acyl-CoAs, which impair insulin-stimulated glucose transport [5,6]. Although selection for increased intramuscular fat content is not expected to impair pig health, it might have consequences on muscle lipid metabolism that need to be determined. Moreover, given the particularly high intramuscular fat content of Duroc pigs, they represent an interesting animal model to study the changes in gene expression that are produced in response to increased fat deposition. We have evaluated the muscle transcriptomic profiles of Duroc pigs displaying divergent fatness phenotypes to gain new insights into these fundamental questions.

## Methods

### Biological samples

Animals came from a commercial Duroc line used for the production of fine quality cured ham and characterised by a high intramuscular fat content. This commercial line was being selected for a compound objective including prolificacy, growth, intramuscular fat and leanness. A population of 350 half-sib castrated males was generated by mating five males with 400 females and selecting one male offspring per litter, as described in Gallardo et al.[7]. A total of 70 phenotypes on growth, fatness, feed efficiency and carcass and meat quality traits were recorded in these animals, including weight, daily food intake, fat deposition measures, and intramuscular fat content and fatty acid composition (C:12-C:22 interval) of *gluteus medius *and *longissimus thoracis et lumborum *muscles [8]. In addition, serum total cholesterol, HDL- and LDL-bound cholesterol and triglycerides were measured in two blood samples taken at 45 and 190 days of age as described in Gallardo et al. [7]. At slaughter, samples of muscle from the *gluteus medius *were collected, snap frozen in liquid nitrogen and stored at -80°C until analysed. The experimental procedures, traits recording and blood sampling were approved by the Ethical Committee of the Institution (IRTA - *Institut de Recerca i Tecnologia Agroalimentàries*).

### Experimental design

**Figure 1:**
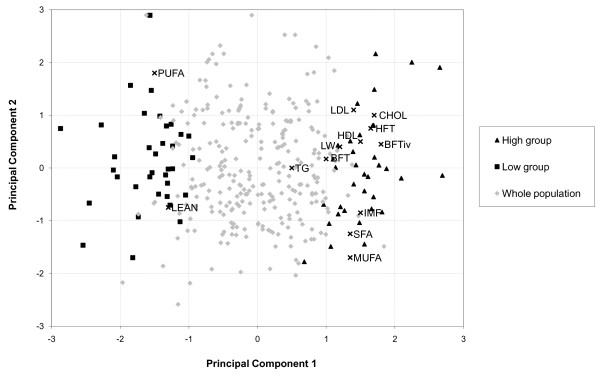
**Graphical plot of the first and second principal components summarising phenotypic variation in the Duroc population**. Summary of phenotype variation on serum lipid, growth and fatness parameters in the Duroc pig population. Relative weight of considered variables and position of animals from the HIGH and LOW groups are represented in the graph. Abbreviations are defined in Table 1.

With the aim of mimicking to some extent the effects of divergent selection for two extreme fatness phenotypes, a principal component analysis (PCA) that allowed synthesising global phenotypic variability recorded in our population was performed by means of the PRINCOMP procedure of SAS (SAS Inst. Inc., Cary, NC). Several preliminary analyses considering the whole set of lipid-related phenotypes (including muscle content of individual fatty acids and several carcass fat measures) allowed us to detect traits with either low variability or displaying high correlations with other ones. These traits did not contribute significantly to the phenotypic variance explained by the principal components and were discarded from further analyses. Finally, a subset of 13 measured traits (Table 1) were considered as the most relevant descriptors of total population variability related to lipid metabolism and lipid content of *gluteus medius *muscle, and were included in the PCA. The first principal component (see Table 1 for coefficients) explained 30.7% of the global phenotypic variance of these traits and was used to rank the animals. Pigs from the extremes of the ranking were selected to create the HIGH (n = 35) and LOW (n = 35) groups, as described in Figure 1. More details about the PCA results and phenotypic variation of the HIGH and LOW groups with divergent phenotypes for fatness traits is provided in the results section.

**Table 1:** Total population, HIGH and LOW group mean values ± standard error for the traits used in the selection index

	Weight in PC1^†^	Population *n = 350*		HIGH group *n = 35*		LOW group *n = 35*	
*Carcass traits*							
**LW **- Live weight (Kg)	0.218	122.08	± 0.74	127.30	± 1.94	111.94	± 2.66
**BFTiv **- Backfat thickness (*in vivo*) (mm)	0.342	23.58	± 0.26	27.26	± 0.69	19.00	± 0.64
**BFT **- Backfat thickness 3^rd^-4^th ^ribs (mm)	0.200	38.31	± 0.60	47.43	± 1.84	32.49	± 1.71
**HFT **- Ham fat thickness (mm)	0.328	25.71	± 0.19	28.47	± 0.41	20.94	± 0.61
**LEAN **- Lean %	-0.265	40.86	± 0.24	38.26	± 0.79	45.94	± 0.71
*Meat quality traits (gluteus medius)*							
**IMF **- % Intramuscular fat	0.304	5.17	± 0.10	7.46	± 0.39	3.58	± 0.15
**SFA **- % Saturated fatty acids	0.276	36.46	± 0.10	38.55	± 0.25	34.65	± 0.21
**PUFA **- % Polyunsaturated fatty acids	-0.315	20.62	± 0.32	14.37	± 0.52	27.35	± 0.81
**MUFA **- % Monounsaturated fatty acids	0.275	42.92	± 0.27	47.08	± 0.45	38.00	± 0.80
*Serum lipid levels - 190 days*							
**CHOL **- Total cholesterol (mg/dl)	0.335	125.15	± 1.40	157.60	± 5.02	104.03	± 2.70
**HDL **- HDL-cholesterol (mg/dl)	0.285	51.66	± 0.54	61.63	± 1.27	43.26	± 1.60
**LDL **- LDL-cholesterol (mg/dl)	0.266	63.12	± 1.09	82.65	± 4.70	50.58	± 2.53
**TG **- Triacylglycerides (mg/dl)	0.101	51.37	± 1.25	66.43	± 4.46	47.66	± 4.75

The weight of each trait in the first principal component (PC1) is also indicated.

**^†^**PC1 - weight of each trait in the first principal component.

### RNA isolation and microarray hybridisation

Samples of *gluteus medius *were ground with mortar and pestle in liquid nitrogen and homogenised with a mechanical rotor. RNA was isolated by the acid phenol method [9] using the RiboPure kit (Ambion, Austin, TX). RNA was quantified in a Nanodrop ND-1000 spectrophotometer and checked for purity and integrity in a Bioanalyzer-2100 (Agilent Technologies, Inc., Santa Clara, CA).

Seventy total RNA samples from the HIGH (n = 35) and LOW (n = 35) groups were individually hybridised on GeneChip Porcine Genomic arrays (Affymetrix, Inc., Santa Clara, CA). A randomised list of the samples was generated to assign hybridisation order to the 70 RNA samples. Total RNA was used to synthesize double stranded cDNA using the One Cycle cDNA Synthesis Kit (Affymetrix, Inc.) which incorporates a T7 RNA polymerase promoter. Biotin-labelled antisense cRNA was obtained using the same kit starting with 5 μg of total RNA and the oligo-(dT) primer 5'-GGCCAGTGAATTGTAATACGACTCACTATAGGGAGGCGG-(dT)_24_. cRNAs were purified with the GeneChip Sample Cleanup Module (Affymetrix, Inc.) and then 20 μg of cRNA were fragmented at 94°C for 30 min in 40 μl of 40 mM Tris-acetate pH 8.1, 100 mM KOAc, 30 mM Mg(OAc)_2_, checked using the Bioanalyzer 2100 (Agilent Technologies, Inc.) and added to a hybridisation cocktail containing control oligonucleotide B2 (50 pM) and Eukaryotic Hybridization controls (BioB, BioC, BioD, Cre) at 1.5, 5, 25 and 100 pM final concentration respectively from the GeneChip Eukaryotic Hybridisation Control Kit (Affymetrix, Inc.), herring sperm DNA (0.1 mg/ml) and acetylated BSA (0.5 mg/ml). The GeneChip Porcine Genome Array was equilibrated to RT and prehybridised with 1× hybridisation buffer (100 mM MES, 1 M [Na+], 20 mM EDTA, 0.01% Tween 20) at 45°C for 10 min with rotation. The hybridisation cocktail was heated to 99°C for 5 min in a heat block, transferred to 45°C for 5 min, added to the arrays and hybridised at 45°C for 16 hours with rotation in the Affymetrix GeneChip Hyb Oven 640 (Affymetrix, Inc.). GeneChips were washed and labelled with streptavidin phycoerythrin in the Fluidics Station 450 (Affymetrix, Inc.) using the protocol EukGE-WS2-v5 provided by Affymetrix. GeneChips were finally scanned in an Agilent G3000 GeneArray Scanner (Agilent Technologies, Inc.).

### Quality control of expression data

The "Affy" and "Sympleaffy" packages from the Bioconductor project [10] were used to implement a set of quality control metrics recommended by Affymetrix to assess the quality of RNA samples and their subsequent labelling and hybridisation steps [11]. These include comparison of the background signal, intensity scaling factor, percentage of genes called, and 3'/5' intensity ratio of control probes in all arrays. Quality control analysis performed with raw intensity data resulted in the identification of two arrays with problems in the labelling efficiency of the 5' *vs *3' control probes. Therefore, data from these two arrays were discarded from further analysis, reducing the number of data available to 68 arrays (34 per group).

### Class comparison analysis on expression data

Data pre-processing, normalisation and class comparison analysis were carried out with the BRB-ArrayTools software version 3.7.1 [12], which is available online at http://linus.nci.nih.gov/BRB-ArrayTools.html, as follows: microarray data normalisation was performed using the gcRMA algorithm, which corrects the intensity of each probe by its GC content [13]; genes showing minimal variation across the set of arrays were excluded from the analysis, that is, only genes displaying more than 20% of expression values over ± 1.5 times the median expression of all arrays were used for further analysis. From the total 23937 spots of the array, 4299 spots passed these filtering conditions. For each probe, expression fold-change was calculated as the ratio between median values in both groups.

Class comparison was performed with a dataset of 68 samples of *gluteus medius *from the HIGH (n = 34) and LOW (n = 34) groups. A global two-group t-test with a random variance model was performed. This type of t-test is an improvement over the standard one-gene *t*-test, as it permits sharing information about within-class variation among genes without assuming that all genes have the same variance [14,15]. By using these variance estimates, this method gains degrees of freedom over the standard t-test, providing greater sensitivity with no loss in specificity [14]. For each probe, the significance p-values of the group effect were calculated based on 100,000 random permutations, and the nominal significance level of each univariate test was restricted to p < 0.01 [12,14]. Additionally, a multiple test correction was performed by restricting the FDR to < 0.05 [16]. The significance level of the whole experiment was also calculated by data permutation: for each permutation, the p-values were re-computed and the number of genes significant at the 0.01 level of the permutation p-value was annotated.

### Gene Ontology-based analysis

We performed a functional categorization of our resulting list of differentially expressed genes using Gene Ontology (GO) information. To annotate the probes in the Affymetrix array we used the latest annotation file available [17]. First, Database for Annotation, Visualization and Integrated Discovery (DAVID, http://david.abcc.ncifcrf.gov) was employed to explore functional class scoring in the resulting gene list by means of GO term enrichment analysis [18]. Significance levels were calculated following a modification of Fisher's exact test. A multiple testing-corrected p-value was also calculated using Benjamini and Hochberg algorithm [16], and only GO terms with Benjamini-corrected p < 0.1 were considered. DAVID was additionally used to explore the biological pathways enriched in the resulting gene list, using information from each individual gene and computing a total over-representation value for each pathway represented in the Kyoto Encyclopaedia of Genes and Genomes (KEGG, http://www.genome.jp/kegg) and BioCarta http://www.biocarta.com.

As a complementary approach, Ingenuity Pathway Analysis, Ingenuity Systems (IPA, http://www.ingenuity.com) bioinformatic tools were employed to explore the distribution of differentially expressed genes in well reported canonical signal transduction or metabolic pathways integrating fold-change data from the microarray experiment and gene-to-gene or protein-to-protein interaction information generated from the literature databases. In this approach, statistical significance of pathway overrepresentation was established with respect to a null distribution constructed by permutations.

### Systems biology studies

We have used the Pathway Express program [19] to identify pathways affected by the list of genes differentially expressed between groups. This program integrates topological information regarding the position of genes in the metabolic and gene signalling pathways defined by KEGG and the multiple roles that these genes might have depending on the pathway they belong to. This program calculates a Perturbation Factor for each gene, which is computed by normalising the expression ratio with information about its topology in the pathway (i.e., number of genes interacting with it and type of interaction) [19]. In each pathway, Perturbation Factors from each individual gene are combined to calculate an Impact Factor, which is normalised according to the total number of genes in the pathway [19]. The complete list of genes in the array was used as a reference set in the analysis.

### Quantitative real time PCR

Quantitative real time PCR (qPCR) was used to validate relevant genes differentially expressed between the two groups of animals with extreme values for lipid metabolism traits. With this aim, a subset of the 10 most extreme animals from each group was selected. Total RNA samples were retrotranscribed with random hexamers and SuperScript III retrotranscriptase (Invitrogen, Carlsbad, CA) following manufacturer's instructions. cDNA was diluted 1:10 in DEPC-treated H_2_O prior to qPCR analysis. Primers and TaqMan probes (Additional file 1) were designed with the Primer Express software (Applied Biosystems, Foster City, CA) using pig sequences obtained by performing a BLAST search with the array probe sequence. For each gene, a standard curve was generated by amplifying serial dilutions of a control ss-cDNA to check for linearity between initial template concentration and Ct values. Quantitative real-time PCR assays were carried out in triplicate in an ABI-7500 device (Applied Biosystems) in a final volume of 5 μl containing 1× Power SYBRgreen Master mix (Applied Biosystems) and 200 nM of each primer. For the two reference genes (*HPRT *and *RPL32*), PCR reactions contained 300 nM of each primer, 200 nM TaqMan probe and 1× Universal Taqman Master Mix (Applied Biosystems). The following thermal profile was used for all reactions: 10 min at 95°C, 40 cycles of 15 s at 93°C and 1 min at 60°C, followed by a quick denaturation at 95°C for 5 min plus a slow ramp to 30°C to generate a dissociation curve to control the specificity of the amplified product. In order to quantify and normalise the expression data we used the ΔΔCt method [20] using the mean Ct value from the two reference genes and the Ct values for each test gene. For each gene, expression values between groups were compared with a t-test and differences were considered significant at p < 0.05. The correlation analysis between qPCR and microarray expression data was performed using the CORR procedure of SAS (SAS Inst. Inc.).

## Results

### Phenotypic variation in the two groups with highly divergent phenotypes for fatness traits

A principal component analysis was performed in order to summarise the global variability of traits strongly related to lipid deposition and composition. Figure 1 displays the relative weight of each variable in the two first principal components, along with the relative position of animals selected for the HIGH and LOW groups. The first principal component explained 30.7% of total variability, whereas the second and following principal components accumulated less than 16% of phenotypic variability. The first principal component (Figure 1) grouped several fatness (fat thickness) measures and serum cholesterol values, together with intramuscular fat, saturated and monounsaturated fatty acids content in *gluteus medius*, while lean percentage and *gluteus medius *polyunsaturated fatty acids content were closely located at the other extreme of the axis. It is worth mentioning that intramuscular fat, monounsaturated and saturated fatty acids content were consistently grouped together in the three first principal components, in concordance with phenotypic correlations and physiological relationships among them [21], whereas polyunsaturated fatty acids always had an opposite relative weight. We finally used the first principal component as a classification index to rank all animals in the experiment (Table 1). Pigs at the higher and lower 10% of the ranking were used to generate the HIGH (n = 35) and LOW (n = 35) groups. For all traits, phenotypic means differed significantly between groups and also with regard of the population mean (Table 1). The highest differences were observed in traits directly related to fatness and muscular fat characteristics. At the top extreme of the ranking, the classification index positioned animals with increased subcutaneous and intramuscular fat deposition. These two traits are important in the dry-cured ham manufacturing process. Monounsaturated and polyunsaturated fatty acids percentages were weighted in opposite directions, thus favouring HIGH group displaying higher oleic and lower polyunsaturated fatty acid contents in the final product. This is an advantageous feature because it reduces the rancidity potential of meat.

### Differential expression between HIGH and LOW groups

A total of 1060 probes were found differentially expressed between animals from the HIGH *vs *LOW groups at a nominal p < 0.01 and restricting the FDR to 0.05 (Additional file 2). A very high overall significance level of the experiment was obtained (p < 10^-7^), confirming differences in the expression pattern of the *gluteus medius *muscle of these two groups of animals. From the 1060 probes, most of them (n = 839) were overexpressed in the HIGH group while only 221 showed higher expression levels in the LOW group. Expression fold-change between groups ranged from 0.38 (repressed in HIGH) to 5.58 (overexpressed in HIGH) and only 388 probes displayed more than 1.5-fold change between groups. The actual range of fold-change values depends on the data pre-processing and normalisation. Indeed, the most commonly used algorithms (MAS5, RMA and gcRMA) compress data in different ways, thus affecting the proportion of large fold-changes between groups simply due to the greater or smaller ranges of expression intensities across replicate values [14]. In this sense, we used here the gcRMA algorithm which moderately compresses data, less than RMA but more than MAS5. Since fold-change ranges vary depending on the algorithm employed, establishing a cutting threshold of 1.5- or 2.0-fold as biologically significant is meaningless. Consequently, we used the complete list of differentially expressed probes in the subsequent analyses. From the list of 1060 probes, 10 were not annotated, 94 were assigned to EST or ORF sequences, and 956 were assigned to genes (corresponding to 836 single genes; Additional file 2). In a microarray, some genes are represented by several probes to evaluate the consistency of gene expression measurements. In our study, 442 single genes showed differential expression for the whole set of probes (100% of consistency across probes), while for the remaining 394 single genes, only a subset of the probes in the array were found to be differentially expressed between groups. The microarray data related to all samples have been deposited in the Gene Expression Omnibus (GEO) public repository (GEO accession number: GSE19275).

### Ontological analysis of differentially expressed genes

GO term annotations were used to perform a functional categorisation of the resulting list of genes generated in the class comparison analysis. The three GO categories (biological process, metabolic function and cell component) were explored with DAVID bioinformatic tools for overrepresentation of specific GO terms. Significant results (at Benjamini-corrected p < 0.1) are summarised in Additional file 3. The most relevant results were obtained under the Biological Process category. Within this category, the top of the list gathered several terms related to "RNA processing", while spread over the list there were a variety of general "metabolic process" terms. Interestingly, four specific GO terms related to "lipid metabolism" appeared in the middle of the list, evidencing that a substantial fraction of genes were directly associated with traits under study.

### Metabolic pathways and gene networks affected by the differentially expressed genes

The analysis of metabolic pathways and gene transactivation networks where the differentially expressed genes play a prominent role is essential to extract biological information and conclusions from a microarray experiment. In order to extract most information from our between-groups gene expression data, results from three exploration tools were compared.

**Figure 2:**
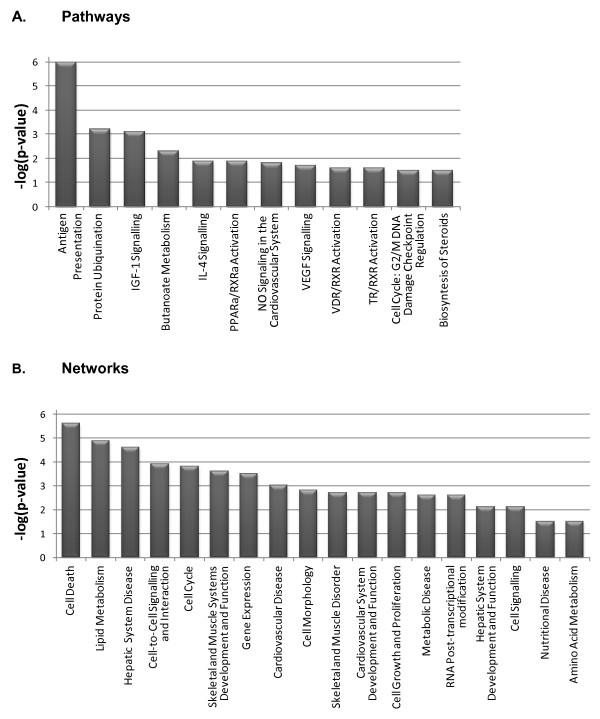
**Functional categorisation analysis of the list of genes differentially expressed between the HIGH and LOW groups obtained with IPA**. A: List of most significant pathways represented in the list of genes (p < 0.01). B: List of most significant networks affected by the joint effect of represented pathways (p < 0.01).

First, the IPA software was used to survey the pathways overrepresented in our gene list. A total of 12 pathways were above the significance threshold (Figure 2A). Strikingly, the *Antigen presentation pathway *was the most significant route. In addition, several gene transactivation cascades involving the RXR factor were also included (Figure 2A). Besides, the *IGF-1 signalling pathway*, which determines the fat *vs *muscle growth, resulted very significant (p < 0.001) in this analysis. As regards the regulatory networks (Figure 2B), several lipid metabolism-, energy uptake- and muscle growth and function-related pathways were amongst the most relevant networks represented by the list of differentially expressed genes.

A second exploration tool, DAVID Functional Annotation tool, identified none of the BioCarta pathways as affected by the list of differentially expressed genes. In contrast, 12 KEGG pathways (p < 0.1; Table 2) were identified and, remarkably, eight of them played a relevant role in lipid metabolism or muscle and/or adipose tissue differentiation (arrows in Table 2).

**Table 2:** DAVID analysis of pathways significantly enriched in the list of differentially expressed genes

KEGG id	KEGG pathway name	Genes (n)	Genes	Fold^†^	p-value*
►01040	Polyunsaturated fatty acids biosynthesis	7	*SCD, TECR, ELOVL5, ELOVL6, FASN, PTPLB, ACAA1*	8.3046	0.00001
05222	Small cell lung cancer	15	*LAMB1, PIK3CA, LAMA5, LAMA2, CYCS, BIRC3, MAX, PIK3R1, ITGB1, COL4A1, ITGAV, CKS1B, LAMC2, BIRC4, RXRG*	3.2728	0.0001
04612	Antigen processing and presentation	12	*HSPA2, HSPA4, HLA-A, PSME1, HSP90AA1, TAPBP, HLA-DRA, B2M, HLA-G, CD74, NFYA, HLA-DMB*	2.8473	0.0027
04510	Focal adhesion	21	*PIK3CA, LAMB1, LAMA5, VEGFA, LAMA2, RAP1A, COL5A3, BIRC3, PIK3R1, ITGB1, IGF1R, COL4A1, ITGB3, THBS4, ITGAV, CAV1, LAMC2, EGFR, BIRC4, ROCK1, ROCK2*	2.0031	0.0034
►04910	Insulin signalling pathway	15	*PIK3CA, ACACA, PPARGC1A, RHOQ, EIF4E, LIPE, PIK3R1, PRKAR2B, IRS2, PPP1R3B, FASN, SORBS1, PRKAB2, IRS1, PRKAG2*	2.1248	0.0097
►03320	PPAR signalling pathway	9	*SCD, OLR1, FABP4, PPARG, ADIPOQ, ACAA1, RXRG, PPARD, SORBS1*	2.3727	0.0334
►04512	ECM-receptor interaction	10	*LAMB1, LAMA5, THBS4, ITGAV, LAMA2, COL5A3, LAMC2, ITGB1, COL4A1, ITGB3*	2.1570	0.0396
►04070	Phosphatidylinositol signalling system	9	*PIK3CA, ITPR1, DGKZ, SYNJ1, PLCD4, FN3K, PIK3R1, PIK3C2A, DGKG*	2.2479	0.0444
04150	mTOR signalling pathway	7	*ULK2, PIK3CA, VEGFA, EIF4E, PIK3R1, ULK3, RICTOR*	2.6054	0.0488
►04930	Type II diabetes mellitus	6	*PIK3CA, IRS2, CACNA1C, ADIPOQ, PIK3R1, IRS1*	2.5309	0.0844
►04920	Adipocytokine signalling pathway	8	*IRS2, PPARGC1A, ADIPOQ, ADIPOR2, RXRG, IRS1, PRKAB2, PRKAG2*	2.0802	0.0862
►04350	TGF-beta signalling pathway	9	*BMP5, FST, THBS4, CUL1, ZFYVE16, TGFB2, ROCK1, SMAD5, ROCK2*	1.8982	0.0978

**^†^**Fold enrichment of the KEGG pathways in the list of differentially expressed genes with regard to the background dataset; *p-value from Fisher's exact test. Arrows indicate pathways relevant to muscle/adipose tissue function.

A third method, Pathway Express [19], was used to explore the existence of signalling networks connecting differentially expressed genes. A total of 19 pathways were inferred from the list of genes with different mRNA levels in both groups (p < 0.05; Table 3). Among these, we found the 12 KEGG pathways previously described by DAVID's Functional Annotation tool but with important differences in the ranking of relevance. A KEGG diagram showing genes in each pathway is presented in the Additional file 4A to H. Many of the significant pathways were very relevant to the muscular and/or adipose physiology (arrows in Table 3). Like in IPA, the pathway with the highest impact factor was *Antigen presenting and processing*, followed then by *Phosphatidylinositol signalling *and *Biosynthesis of unsaturated fatty acids*. Regarding the latter, seven out of the 23 genes in this route, which encode enzymes directly involved in the elongation and desaturation of fatty acids, were differentially expressed between the HIGH and LOW groups (Table 3, Additional file 4C). Only one of them (*GPSN2 *- a reductase that participates in the desaturation of long and very long chain fatty acids) is overexpressed in the LOW group whereas the other six have higher levels in the HIGH group. The *Insulin signalling *and *Type II diabetes *pathways (Additional file 4D and E), which are deeply associated with glucose metabolism, were differentially expressed in both groups. Among genes that take part in (or are connected with) the insulin signalling cascade, one should emphasise the following groups, all of them expressed at higher levels in the HIGH group: (1) *mitogen-activated protein kinase *(*AMPK*), *acetyl-coA carboxylase α *(*ACACA*) and *fatty acid synthase *(*FASN*) genes that have a lipogenic role; (2) *PKA *and *hormone-sensitive lipase (LIPE*) genes which display a lipolytic action; (3) *TBC1 domain family, member 1 *gene (*TBC1D1*), which plays a role in the translocation of the glucose transporter 4 (GLUT4) from the endoplasmic reticulum to the cell membrane in response to the insulin signal; and (4) *peroxisome proliferator-activated receptor-γ coactivator 1 gene *(*PPARGC1A*), an adapter of the PPAR family of nuclear receptors, which takes part in the transactivation of effector genes. In addition, it is worth to mention the differential expression of the *insulin receptor substrate 2 *(*IRS2*) gene, first effector downstream the insulin receptor. The effect on this substrate is, therefore, directly transmitted to the rest of the cascade.

**Table 3:** Pathway Express analysis of metabolic pathways and gene networks significantly affected by differentially expressed genes

KEGG pathway-id	KEGG pathway name	IF^†^	input/pathway genes	pathway genes on chip	p-value*
04612	Antigen processing and presentation	61.066	15/88	45	1.87E-25
►04070	Phosphatidylinositol signalling system	18.350	10/77	60	2.08E-07
►01040	Biosynthesis of unsaturated fatty acids	10.791	7/23	18	2.43E-04
05222	Small cell lung cancer	8.686	14/87	71	1.64E-03
04510	Focal adhesion	7.117	20/199	169	6.58E-03
05332	Graft-versus-host disease	6.966	6/42	22	7.52E-03
►04910	Insulin signalling pathway	6.430	16/138	114	1.20E-02
05310	Asthma	6.323	5/30	17	1.31E-02
04520	Adherents junction	5.957	6/75	69	1.80E-02
04514	Cell adhesion molecules (CAMs)	5.952	13/133	95	1.81E-02
05330	Allograft rejection	5.813	6/38	26	2.04E-02
►04930	Type II diabetes mellitus	5.556	6/44	33	2.53E-02
►04512	ECM-receptor interaction	5.211	10/87	72	3.39E-02
05320	Autoimmune thyroid disease	4.955	6/53	31	4.20E-02
04940	Type I diabetes mellitus	4.955	6/44	31	4.20E-02
04150	mTOR signalling pathway	4.932	7/51	42	4.28E-02
►04920	Adipocytokine signalling pathway	4.779	8/72	64	4.66E-02
►03320	PPAR signalling pathway	4.684	9/69	61	4.75E-02
►04350	TGF-beta signalling pathway	4.662	9/89	74	4.95E-02

**^†^**Impact Factor; *p-value from Fisher's exact test. Arrows indicate pathways relevant to muscle/adipose tissue function.

The *PPAR signalling *pathway is one of the most relevant routes during the process of adipocyte tissue development, differentiation and activation of lipogenesis (Additional file 4F). In the HIGH group we observed increased mRNA levels of *peroxisome proliferator-activated receptor δ *(*PPARD*), *γ *(*PPARG*) and *PPARGC1 *while *retinoid × receptor γ *(*RXRG*) mRNA was downregulated. Moreover, the *fatty acid binding protein 4 *(*FABP4*) gene, a key player in the uptake of fatty acids, exhibited higher mRNA levels in pigs of the HIGH group. We also observed changes in the *Adipocytokine signalling pathway *(Additional file 4G), particularly the mRNA levels for *ADIPOQ *and *adiponectin receptor 2 *(*ADIPOR2*) were overexpressed in the HIGH group (Additional file 4G) alongside with *AMPK*, *RXRG*, *PPARGC1A *which indirectly affect the oxidation and degradation of lipids in mitochondria through the *carnitine palmitoyltransferase 1 *gene (*CPT1*).

Two other pathways related to myocyte growth and differentiation happened to be affected by the genes differentially expressed between the HIGH and LOW groups. First, the *Extracellular matrix interaction *pathway (Additional file 4H), which consists of a complex mixture of structural and functional macromolecules (integrins, proteoglycans, CD36, and other cell-surface-associated components) with an important role in tissue and organ morphogenesis and function [22]. From the genes that take part in this pathway, we have identified expression changes in *α *and *β integrins*, *collagen *and *transforming growth factor β *(*TGFβ*) *receptor *genes. The second pathway of interest was the *TGFβ signalling *route (Additional file 4I) that participates in many cellular processes like cell differentiation, proliferation and apoptosis. The *TGFβ *factor differentially expressed in the HIGH group was *TFGB2*, whose role in the muscular or adipose tissue development is not currently known.

### Quantitative PCR validation of microarray results

**Figure 3:**
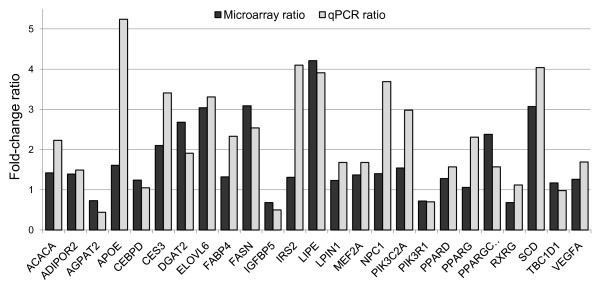
**Comparison of microarray and qPCR expression ratios (HIGH vs LOW groups) for the list of selected genes**.

The information relative to the gene ontology and pathways analysis was used to select 31 genes with a relevant function in muscle and adipose physiology and lipid metabolism (Additional file 5). The selection was also based on literature and database searches for each individual gene from the resulting a 879-genes list. In this way, genes functionally related to muscle and fat cell metabolism were selected to be validated by qPCR in a subset of 10 animals from each group. In order to confirm the probe annotation information, we first conducted a BLAST search with the sequences used by Affymetrix to generate the microarray probes. Three of the 31 probes analysed were wrongly annotated and were, therefore, excluded from further analysis. Efficient qPCR assays were established for 25 out of the 28 remaining genes. For all 25 genes (Table 4), the fold-change ratios between HIGH and LOW groups were consistent in both assays except for *TBC1D1 *and *RXRG *genes. In addition, in most cases the expression ratios were higher in the qPCR experiment than in microarray based analyses (Figure 3) [23]. A high level of correlation (ranging from 0.48 to 0.96; p < 0.05) was observed between qPCR and Affymetrix microarray data for all genes except for *TBC1D1 *and *RXRG *(Table 4).

**Table 4:** Validation of microarray expression data by quantitative real-time PCR for 25 selected genes

GENE		MICROARRAY		qPCR		Correlation	
		n = 68(34H *vs *34L)		n = 20(10H *vs *10L)			
		Ratio^†^	p-value	Ratio^†^	p-value	r*	p-value
**ACACA**	↑	**1.42**	**0.003**	**2.23**	**0.047**	**0.77**	**0.0002**
ADIPOR2	↑	1.39	0.001	1.49	0.354	0.59	0.0108
**AGPAT2**	↓	**0.73**	**0.01**	**0.44**	**0.043**	**0.60**	**0.0099**
**APOE**	↑	**1.61**	**0.001**	**5.24**	**0.017**	**0.73**	**0.0006**
CEBPD	↑	1.24	0.004	1.05	0.078	0.69	0.0016
**CES3**	↑	**2.1**	**0.000003**	**3.41**	**0.008**	**0.83**	**< 0.0001**
**DGAT2**	↑	**2.68**	**0.001**	**1.91**	**0.048**	**0.83**	**< 0.0001**
**ELOVL6**	↑	**3.04**	**0.005**	**3.31**	**0.042**	**0.88**	**< 0.0001**
**FABP4**	↑	**1.32**	**0.0002**	**2.33**	**0.037**	**0.92**	**< 0.0001**
**FASN**	↑	**3.09**	**0.0008**	**2.54**	**0.046**	**0.88**	**< 0.0001**
**IGFBP5**	↓	**0.68**	**0.0007**	**0.50**	**0.025**	**0.83**	**< 0.0001**
**IRS2**	**↑**	**1.31**	**0.001**	**4.1**	**0.012**	**0.53**	**0.0231**
**LIPE**	↑	**4.21**	**0.000002**	**3.91**	**0.001**	**0.76**	**0.0002**
**LPIN1**	↑	**1.23**	**0.008**	**1.68**	**0.005**	**0.68**	**0.0018**
**MEF2A**	↑	**1.37**	**0.0002**	**1.68**	**0.037**	**0.63**	**0.0051**
**NPC1**	**↑**	**1.4**	**0.00006**	**3.69**	**0.018**	**0.48**	**0.0437**
**PIK3C2A**	**↑**	**1.54**	**0.009**	**2.98**	**0.021**	**0.68**	**0.0020**
**PIK3R1**	↓	**0.72**	**0.01**	**0.70**	**0.020**	**0.48**	**0.0424**
**PPARD**	↑	**1.28**	**0.008**	**1.57**	**0.021**	**0.53**	**0.0248**
**PPARG**	↑	**1.06**	**0.005**	**2.31**	**0.041**	**0.69**	**0.0016**
PPARGC1A	↑	2.38	0.004	1.57	0.152	0.96	< 0.0001
RXRG	↓	0.68	0.002	1.12	0.621	-0.26	0.2911
SCD	↑	3.07	0.004	4.04	0.156	0.96	< 0.0001
TBC1D1	↑	1.17	0.0002	0.98	0.552	-0.16	0.5287
**VEGFA**	↑	**1.26**	**0.009**	**1.69**	**0.032**	**0.48**	**0.0444**

Expression ratios and p-values are indicated for both assays and correlation coefficients between qPCR and microarray results are also shown.

H = HIGH group; L = LOW group; **^†^**Expression ratios between H and L groups; *correlation coefficients between qPCR and microarray results; in **bold**, genes whose difference in expression between groups has been validated by qPCR. Arrows up and down indicate gene overexpression and downregulation in the HIGH group, respectively.

All together, validation of microarray data by qPCR revealed a high correspondence between both analyses, confirming differential expression for 19 out of 25 genes tested (p < 0.05). The significance level of the differences between HIGH and LOW groups tended to be greater in the microarray experiment, which might be due to the higher background noise of the qPCR assay.

## Discussion

The ultimate goal of selection is to fix a set of genotypes that are advantageous from a production, economic or nutritional point of view [24]. This might involve significant changes in the physiology and metabolism of individuals under selection [25]. In pigs, traditional selection has been focused to obtain leaner individuals but this practice has had detrimental consequences on meat quality due to a reduction in intramuscular fat and a parallel increase in polyunsaturated fatty acids [26], which are highly susceptible to lipid oxidation [1]. Genetic and phenotypic correlations suggest that selection for higher intramuscular fat (as a way to improve the palatability and juiciness of meat) might increase fatness, serum lipids and meat monounsaturated fatty acids and saturated fatty acids contents [3]. In summary, selection for increasing either leanness or intramuscular fat might lead to two extreme phenotypes with divergent muscular metabolic profiles. We have been able to capture these two phenotypic classes (HIGH and LOW groups) in a Duroc commercial population that had been selected aiming to increase intramuscular fat while maintaining carcass fatness. The utilization of a PCA approach seems to have been particularly efficient in detecting individuals that are different for a global multitrait phenotype rather than for a particular trait, as supported by the high overall significance achieved in the differential expression experiment. In the next sections we will discuss changes of muscle gene expression that are associated with each one of these composite phenotypes and their physiological and metabolic implications.

### Lipid metabolism

We have observed significant changes in the expression of genes and pathways related to lipid metabolism (Tables 4 and Additional file 1). Fatty acid uptake genes such as *FABP4 *and *Apolipoprotein E *(*APOE*) were upregulated in the HIGH group. Fatty acid-binding proteins are small cytosolic molecules that bind saturated and unsaturated fatty acids. Mice where *FABP4 *expression has been abolished are less susceptible to develop insulin resistance and dislipidemias [27]. APOE remove chylomicron remnants from circulation by interacting with low density lipoprotein receptor (LDLR), LDLR-related protein 1 (LRP1) and other receptors thus favouring lipid deposition [28]. In fact, *APOE *knockout mice have less body fat stores, smaller adipocytes and they are more resistant to diet-induced obesity [28]. This increased expression of fatty acid uptake molecules might represent a compensatory response to the higher levels of serum lipids in the HIGH group (Table 1). *De novo *lipogenesis genes (*i.e*. *ACACA*, *FASN*) were also upregulated (Table 4 and Additional file 1). Malonyl-CoA, a powerful inhibitor of the carnitine/palmitoyl shuttle system for fatty acid β-oxidation and the substrate of FASN [29], is synthesised by ACACA, so increased expression of this enzyme might lead to an accumulation of muscle fat stores [29]. Two other genes promoting lipid deposition, *stearoyl CoA desaturase *(*SCD*) and *diacylglycerol O-acyltransferase 2 *(*DGAT2*), displayed an increased expression in the HIGH group (Tables 4 and Additional file 1). *SCD *plays a key role in the synthesis of monounsaturated fatty acids which, in turn, regulate other fundamental components of lipid metabolism such as sterol regulatory element-binding protein (SREBP) and MAPK. In human, a high SCD activity is associated with a metabolic state favoring hepatic triglyceride accumulation and expansion of adipose triglyceride stores [30]. In consequence, a higher *SCD *expression in the muscle might partition fatty acids towards storage rather than to oxidation [30]. DGAT2 is the main enzyme involved in the synthesis of triglycerides and its increased expression might also favour lipid storage [31]. Similarly, CCAAT/enhancer-binding protein δ (CEBPD) and PPARG, whose expression is upregulated in HIGH pigs, are transcription factors which promote fat deposition [32,33]. This pattern of gene expression suggests that the increased lipid deposition of HIGH pigs is the result of several metabolic processes rather than the consequence of a single biochemical event.

There is substantial evidence that the aforementioned genes are differentially expressed in adipose tissue cells and myocytes (Novartis Gene Expression Atlas, http://biogps.gnf.org/#goto=welcome), while their status in intramuscular adipocytes remains largely unknown [34]. An obvious and unavoidable limitation of our experiment was that we were unable to separate myocytes and intramuscular adipocytes before isolating RNA. The influence of this feature on our expression data is difficult to evaluate. Intramuscular fat (LOW: 3.5%, HIGH: 7.5%) comes from two different sources: small adipocytes localized between muscular fibres and lipid droplets contained within myocytes [34]. This means that the percentage of intramuscular adipocytes cannot be simply calculated based in intramuscular fat content. Moreover, gene expression profile of intramuscular adipocytes is mostly unknown, although there is compelling evidence that in pigs is clearly different from that of subcutaneous and perirenal adipocytes [34,35]. For instance, *FASN*, *PPARG *and *LIPE *mRNAs are 600, 200 and 800 times less expressed in intramuscular than in subcutaneous adipocytes of 160-days-old pigs [34]. Proteomic studies have also evidenced that lipogenic, lipolytic, glycolytic and fatty acid oxidation pathways are clearly downregulated in intramuscular adipocytes when compared with fat cells from other depots [35]. These results suggest that differences in the proportion of intramuscular adipocytes between HIGH and LOW groups might have a minimum impact on the patterns of muscle gene expression. This interpretation is strongly supported by the fact that gene expression changes detected in our experiment are very consistent with a plethora of data obtained in human indicating that fatty acid uptake, lipogenesis and triglyceride synthesis genes are upregulated in the muscle cells of obese individuals [5].

Fat accumulation in the skeletal muscle might be quite harmful for this tissue as well as for the whole organism [6,36]. Obese sedentary individuals have an increased rate of lipid peroxidation and higher levels of certain lipid metabolites, such as diacylglycerol, ceramides and long-chain fatty acids in the myocyte cytoplasm [6]. Both features are well known to promote the development of insulin resistance [6]. In this sense, animals in the HIGH group had upregulated levels of *lipin 1 *(*LPIN1*) mRNA, which induces the synthesis of diacylglycerol from phosphatidic acid [37,38], and of *elongation of very long chain fatty acids-like 5 *and *6 *(*ELOV5, ELOVL6*) mRNA which promote the elongation of fatty acids [39]. These genes might increase the production of metabolites that are well known to have negative effects on insulin metabolism.

In this context, it might be argued that selection for increasing intramuscular fat content might have detrimental consequences on glucose and lipid homeostasis in the skeletal muscle. However, we have obtained substantial evidence of the activation of pathways which might counteract the accumulation of intracellular lipids (Table 4 and Additional file 1). In this way, increased *LIPE *and *CES3 *mRNA expression were detected in the HIGH group. These two lipases are deeply involved in the hydrolysis of triacylglycerols [37]. The remarkable expression differences observed in the HIGH and LOW groups (about 3-4 fold in the qPCR analysis) cannot be explained in terms of adipocyte abundance, but as the consequence of a physiological change in muscle cells. In this regard, the simultaneous upregulation of genes with lipogenic (*ACACA*, *FASN*, *SCD*, *DGAT2*) and lipolytic (*LIPE*, *CES3*) actions would involve the existence of a futile cycle where triacylglycerols are continuously degraded and resynthesised. It has been suggested that, ultimately, intramuscular fat accumulation results from a balance between uptake, synthesis and degradation of triglycerides, rather than the upregulation of a single pathway [40]. Our results seem to point out also in this direction. Moreover, the upregulated *LIPE *and *CES3 *mRNA expression might have a protective effect in the muscle cell by degrading intracellular lipids (such as diacylglycerol) which might otherwise inhibit insulin signalling. Another line of defence against lipid overload would be featured by *ADIPOQ*, *PPARD *and *PPARGC1 *mRNAs, which are upregulated in the HIGH group and promote the catabolism of fatty acids. *PPARGC1A *is an adapter of nuclear receptors which plays a central role in the modulation of gene transactivation in several signalling routes, such as insulin, adipokines and bioactive lipids [41]. In the skeletal muscle, *PPARGC1A *avoids the development of insulin-resistance by increasing the proportion of oxidative fibres and stimulating mitochondrial biogenesis, two features that promote fatty acid oxidation [42]. PPARD, a transcription factor primarily expressed in muscle cells, also enhances fatty acid oxidation and energy expenditure [33]. Finally, adiponectin binding to its receptor also stimulates fatty acid oxidation and decreases triglyceride storage in muscle, which may explain, in part, the insulin-sensitising effect of this hormone [43]. This process relies on the activation of AMPK, a master regulator of cellular energy balance [43]. Activation of AMPK, in turn, depends partly on a phospholipase C/Ca2^+ ^Ca2^+^/Calmodulin-dependent protein-kinase kinase-dependent pathway [44], a feature that agrees well with our observations that *phospholipase C *and *calmodulin 2 *mRNA are upregulated in the HIGH group. Taken together, these results suggest that genes activating the catabolism of fatty acids are upregulated in the HIGH pìgs, although we have not found direct evidence that genes integrated in the *β-oxidation *pathway are differentially expressed in the HIGH and LOW groups. Probably, ADIPOQ and PPARD exert their action by introducing post-translational modifications in the β-oxidation enzymes rather than modifying their levels of expression. Finally, it is worth mentioning that our findings do not agree with the observation that, in human, fatty acid oxidation is reduced in obese individuals [5].

### Insulin signalling and glucose metabolism

We have detected differential expression of genes related to the insulin signalling pathway between the HIGH and LOW groups. *MADS-box transcription enhancer factor 2 polypeptide A *(*MEF2A*, overexpressed in the HIGH group) is abundantly expressed in muscle cells and it binds to the promoter of the *GLUT4 *gene upregulating its expression and glucose uptake [45]. Interestingly, *insulin receptor substrate 2 *(*IRS2)*, one of the two major insulin receptor substrates, was also upregulated in the HIGH group. Impaired function of IRS2 has been related to a defective activation of phosphoinositide 3-kinase (PIK3), a reduction in glucose uptake and insulin resistance in certain studies [46] but not in others [47]. It is also worth mentioning that we have found that *PIK3 regulatory subunit 1 *(*PIK3R1*) and *PIK3 class 2 alpha polypeptide *(*PIK3C2A*) mRNAs are down- and upregulated, respectively. Free, monomeric PIK3R1 can act as a negative regulator of PIK3 signalling downstream the insulin and IGF1 receptors by competing with the PIK3R1/PIK3C heterodimer for binding to tyrosine-phosphorylated IRS1 [48], while PIK3C2A is a subunit of PIK3. As a whole, pigs of the HIGH group, which are fatter than their LOW counterparts, show an mRNA profile compatible with an increased glucose uptake. This does not necessarily mean that the functionality of the molecules involved in this pathway is identical in both groups, because we have not evaluated post-translational changes, which are known to have a critical impact on their activity [49]. At most, we can affirm that glucose uptake and insulin signalling pathways are not downregulated in the *gluteus medius *muscle of HIGH pigs. This observation agrees very well with the fact that *de novo *lipogenesis, which can use glucose as a substrate, is upregulated in this group.

### Immunity

The most striking difference amongst the HIGH and LOW groups was that expression of antigen-presenting molecules was downregulated in the former (Additional file 4A). Interestingly, a significant reduction in the expression levels of genes related to the immunity in muscles from chickens selected for fat content has also been described in other microarray studies [50]. A possible mechanism explaining our results would be related to the increased expression of the *adiponectin receptor 2 *in the muscle cells of HIGH pigs, which increases the synthesis of the antiinflammatory cytokine interleukin (IL) 10, inhibits the synthesis of proinflammatory cytokines, such as IL1β, IL6 and tumor necrosis factor (TNF), and activates AMPK, a kinase that can disrupt *NFκB *transcriptional activation, resulting in downregulation of *MHC *expression [51]. *Stearoyl CoA desaturase *overexpression has also been reported to have antiinflammatory activity through the conversion of palmitate to oleate [52].

### Muscle growth and differentiation

We have observed an upregulation of the *TGFB2 *and *TGFBR3 *mRNAs in the HIGH group (Additional file 2 and 4I). The TGFβ signalling pathway participates in many cellular processes like cell differentiation, proliferation and apoptosis. In the skeletal muscle, TGFβ is a powerful inhibitor of the muscular proliferation and regeneration from satellite cells [53]. For instance, myostatin, which is upregulated by TGFB1, inhibits muscular proliferation [54]. Inactivation of this gene produces an excessive growth of the muscular tissue that results in the "double muscle" phenotype observed in Blue Belgian or Asturiana cattle [55]. Moreover, transforming growth factor β molecules are positive regulators of extracellular matrix proteins expression [56]. In this sense, we have detected increased mRNA levels of *integrins α5 *(*ITGAV*) and *β1 *(*ITGB1*) as well as of several collagen molecules (*COL4A5*, *COL12A1*, *COL4A1*, *COL5A3*). The ability of satellite cells to successfully participate in growth and repair of the skeletal muscle is influenced heavily by surrounding extracellular matrix factors [56]. In this way, the formation of multinucleated myotubes requires the migration of myoblasts, a process regulated by integrins that ensure the adhesion of these precursor cells to the extracellular matrix [56]. Finally, it is worth mentioning that we have detected increased mRNA levels of myogenic factors such as *insulin growth factor-binding protein 5 *(*IGFBP5*) and *ADIPOR2*. These findings might be biologically significant because there are evidences that some level of postnatal muscle growth, relying on the activation, differentiation and proliferation of precursor satellite cells, takes place in pigs [57].

## Conclusions

We have examined the mRNA expression profile of muscle samples obtained from pigs with extreme phenotypes for several fatness parameters. The HIGH phenotype corresponded to pigs with high levels of intramuscular fat, serum lipids and muscle saturated and monounsaturated fatty acid content, while LOW pigs were leaner and showed increased levels of muscle polyunsaturated fatty acids. This approach allowed us to detect striking differences in the expression profile of both groups, with the HIGH group showing upregulated mRNA levels of genes related to lipogenesis, lipolysis and glucose uptake and decreased levels of mRNAs encoding antigen-presenting molecules. As a whole, our data suggest that selection for increasing intramuscular fat content in pigs would not lead to a disruption of the metabolic homeostasis of muscle cells. An evident limitation of our experiment is that mRNA concentrations are not necessarily a faithful reflection of protein levels or activities. In this regard, Maier et al. [58] have reported that correlations between mRNA and protein levels are moderate (r_P _= 0.36) to high (r_P _= 0.76), being influenced by gene-specific (protein half-life, translation efficiency etc.) and technical factors. In consequence data about post-translational changes affecting protein activity or expression as well as information about protein location within the cell would be needed to fully understand how lipid deposition affects muscle physiology in pigs [57].

## Authors' contributions

AC participated in the RNA isolation, initial microarray analysis and validation of the results by qPCR. RQ developed the experimental design and performed the statistical analysis. MA participated in the analysis and interpretation of the data and helped drafting the manuscript. RNP participated in RNA isolation, microarray analysis and ontology analysis. All authors have been involved in drafting and revising the manuscript. All authors read and approved the final manuscript.

## Supplementary Material

Additional file 1 **Sequence of primer sets used to validate microarray results of selected genes by quantitative real-time PCR**. Gene symbol and GenBank accession number for each pig gene are indicated. Putative exon location is based on human gene structure information. Click here for file

Additional file 2 **List and annotation of differentially expressed probes between HIGH and LOW groups**. List and annotation of the 1060 probes differentially expressed between gl*uteus medius *muscle of HIGH and LOW pigs. Parametric, permutation p-values and 5% FDR are indicated. Means and medians of log_2 _intensities are used to calculate ratios of fold-change expression. Click here for file

Additional file 3 **Gene ontology terms**. List of significantly overrepresented gene ontology terms (distributed over the three main family categories) in the list of 1060 probes differentially expressed between *gluteus medius *muscle samples of HIGH and LOW pigs. Parametric p-values are indicated along with Bonferroni, Benjamini and 5% FDR multiple test corrections. Click here for file

Additional file 4 **Graphical representation of KEGG diagrams**. Graphical representation of microarray results over the nine KEGG pathways significantly affected by the genes differentially expressed between the HIGH and LOW groups (p < 0.05). A: Antigen Processing and Presentation; B: *Phosphatidylinositol Signalling*; C: *Biosynthesis of Unsaturated Fatty Acids*; *D*: *Insulin Signalling*; E: *Type II Diabetes Mellitus*; F: *PPAR Signalling*; G: *Adipocytokine Signalling*; H: *ECM-receptor Interaction*; I: *TGF-beta Signalling*. In red, genes overexpressed in the HIGH group. In blue, genes overexpressed in the LOW group. Click here for file

Additional file 5 **List of genes selected for real time qPCR validation**. Genes were selected for their involvement in lipid metabolism or muscle/fat development. A brief summary describing gene function and involved in KEGG pathways is provided. Click here for file
