# Supplementary material for: Muscle transcriptomic profiles in pigs with divergent phenotypes for fatness traits
Source: BMC Genomics. 2010 Jun 11;11:372. doi: 10.1186/1471-2164-11-372 (PMC2894043; doi:10.1186/1471-2164-11-372)
Supplement: Additional file 1 — Sequence of primer sets used to validate microarray results of selected genes by quantitative real-time PCR. Gene symbol and GenBank accession number for each pig gene are indicated. Putative exon location is based on human gene structure information. [file 1471-2164-11-372-S1.PDF]

Additional file 1: Sequence of primer sets used to validate microarray results for selected genes by quantitative real-time PCR. Gene symbol and GenBank accession number for each pig gene are indicated. Putative exon location* is based on human gene structure information.

Gene Symbol	Gene Name	Forward Primer	Exon*	Reverse Primer	Exon*	GenBank
**ACACA**	Acetyl-CoA carboxylase 1	CCCGAGCTGACCGATGG	E1	GAGCGAACACCGTCTTCCTC	E1	NM_001114269
**ADIPOR2**	Adiponectin receptor protein 2	GCCCACCATGCAATGGA	E3	GAGGGATTACTCGCCATCGA	E4	NM_001007192
**AGPAT2**	1-acyl-sn-glycerol-3-phosphate acyltransferase beta	GGGCACACGGAATGACAAC	E4	GGAGTCGTAGAAGGAGGAGAAGCT	E5	NM_001130534
**APOE**	Apolipoprotein E	TGCAGTCCCTGTCTGACCAA	E3	GTAGGCCTTCACCTCCTTCATG	E4	NM_214308
**AQP4**	Aquaporin 4	TGGGAAAACCACTGGATATATTGG	E4	GCCGCCGGCAAGGA	E5	NM_001110423
**CEBPD**	CCAAT/enhancer binding protein delta	CGACTTCAGCGCCTACATAGACT	E1	TGTTGAAGAGGTCAGCGAAGAG	E1	XM_001925928
**CES3**	Liver carboxylesterase 1 precursor (ACAT)	CGCTGCGCATGAAAACG	E4	GTGCTGAAGAATCCCCAGATG	E5	NM_214246
**CIDEA**	Cell death activator CIDE-A	CACACCGCCCAGGTGACT	E5	CAGCATTCGGAGCATGTACGT	E5	NM_001112696
**DGAT2**	Diacylglycerol O-acyltransferase homolog 2	CTTTGAGGAGGGTCCTGGG	E6	GCCTCGGCCGTGGAAG	E6	XM_001925093
**ELOVL6**	Long-chain fatty-acyl elongase family member 6	TGAAATTGGGCAAACACTGTGT	E5	AGGGCACTTTAAGCACCTCTACA	E5	AK232419
**FABP4**	Fatty acid-binding protein, adipocyte	CCAGTGAAAACTTTGATGATTACATGA	E1	TGATGATCAGGTTGGGTTTGG	E2	XM_001927334
**FASN**	Fatty acid synthase	CCTGCCACAACTCCAAAGACA	E12	ATGAAGTAGGAGTGGAAGGCGA	E13	NM_001099930
**IGFBP5**	Insulin-like growth factor binding protein 5 precursor	CGCAAGGGATTCTACAAGAGAAA	E3	TCCACGCACCAGCAGATG	E4	NM_214099
**IRS2**	Insulin receptor substrate-2	AACAGTGTTTTCTTTTTCGGTACGT	E2	TGTCCCAAGAGCCAGAAATATACTT	E2	AY690661
**LIPE**	Hormone sensitive lipase	CGTCAAGAATCCCTTCATGTCA	E9	GCGCAGGCCACAATGTG	E10	NM_214315
**LPIN1**	Lipin 1	CAGAACCTTTTTATGCTGCTTTTG	E18	CAGGGACACTCCCACTTGCT	E19	NM_001130734
**LRP11**	Low-density lipoprotein receptor-related protein 11 precursor	GAACTTAAGATGTTTGTCCTCTACATGTG	E7	ATGGCTATAGGTGACATCCTTCTATATAAA	E7	XM_001928988
**MEF2A**	Myocyte-specific enhancer factor 2A	CCGTTTGGCGGCACAA	E1	CGTTCAGGGAAAAATTGAGATTAAC	E1	NM_001097421

**NPC1**	Niemann-Pick C1 protein precursor	CCTCCCTGTCTTACTCAGTTACATAGG	E24	CGCTCTTGAGTGGCCAGACT	E25	NM_214322
**PIK3C2A**	Phosphoinositide-3-kinase, class 2, alpha polypeptide	CTGAGGAACCCAATGTAGATC	E1	CTAGAGATTCTTAAAAAGGCAGTAA	E1	NM_213939
**PIK3R1**	Phosphatidylinositol 3-kinase regulatory alpha subunit	GGTTGGAGGAGGACCTGAAGA	E12	GCTGTTCATGCGCTTGTCAA	E12	AJ555826
**PPARD**	Peroxisome proliferator activated receptor delta	TGATAGTGACCTGGCTCTCTTCAT	E7	GCCTCCACCTGTGACACGTT	E8	NM_001130241
**PPARG**	Peroxisome proliferator activated receptor gamma	CCTGGCGATATTTATAGCTGTCATT	E7	CTCGATGGGCTTCACATTCA	E8	NM_214379
**PPARGC1A**	Peroxisome proliferator activated receptor gamma coactivator 1 alpha	GAGTCTGAAAGGGCCAAGCA	E10	CAGTTCTGTCCGTGTTGTGTCA	E11	NM_213963
**RXRG**	Retinoic acid receptor RXR-Gamma	AACCAAAGACGGAATCCTATGG	E5	GTTGGTGACAGGGTCATTCGT	E6	NM_001130213
**SCD**	Stearoyl-coA desaturase	CCACTGTGCCACTGACTTGCT	E6	CCCATTTTAACAGAAGGAAAACTGA	E6	NM_213781
**TBC1D1**	TBC1 domain family member 1	GCAGCTTACGCAAACAGAACCT	E19	AGGCTCTGGATCCTCCCATT	E20	AK237784
**VEGFA**	Vascular endothelial growth factor A precursor	GTGCCCACTGAGGAGTTCAAC	E3	TCCTATGTGCTGGCCTTGGT	E4	NM_214084
**HPRT**	Hypoxanthine phosphoribosyltransferase 1	AAGATGGTCAAGGTTGCAAGCT	E6	ATTTCAAATCCAACAAAGTCTGGTCTA	E7	NM_001032376
		HPRT_Taqman probe: JOE-TGGTGAAAAGGACCCCTCGAAGTGTTG-TAMRA			E6/E7	
**RPL32**	Ribosomal protein L32	CACCAGTCAGACCGATATGTCAA	E1	CGCACCCTGTTGTCAATGC	E2	NM_001001636
		PRL32_Taqman probe: Cy5-TAAGCGGAACTGGCGGAAACCCA-BHQ			E1/E2	
