# Supplementary material for: Muscle transcriptomic profiles in pigs with divergent phenotypes for fatness traits
Source: BMC Genomics. 2010 Jun 11;11:372. doi: 10.1186/1471-2164-11-372 (PMC2894043; doi:10.1186/1471-2164-11-372)

Additional file 4. Graphical representation of KEGG diagrams. Graphical representation of microarray results over the nine KEGG pathways significantly affected by the genes differentially expressed between the HIGH and LOW groups ($p<0.05$). A: Antigen Processing and Presentation; B: *Phosphatidylinositol Signalling*; C: *Biosynthesis of Unsaturated Fatty Acids*; *D*: *Insulin Signalling*; E: *Type II Diabetes Mellitus*; F: *PAAR Signalling*; G: *Adipocytokine Signalling*; H: *ECM-receptor Interaction*; I: *TGF-beta Signalling*. In red, genes overexpressed in the HIGH group. In blue, genes overexpressed in the LOW group.

Additional file 4A.

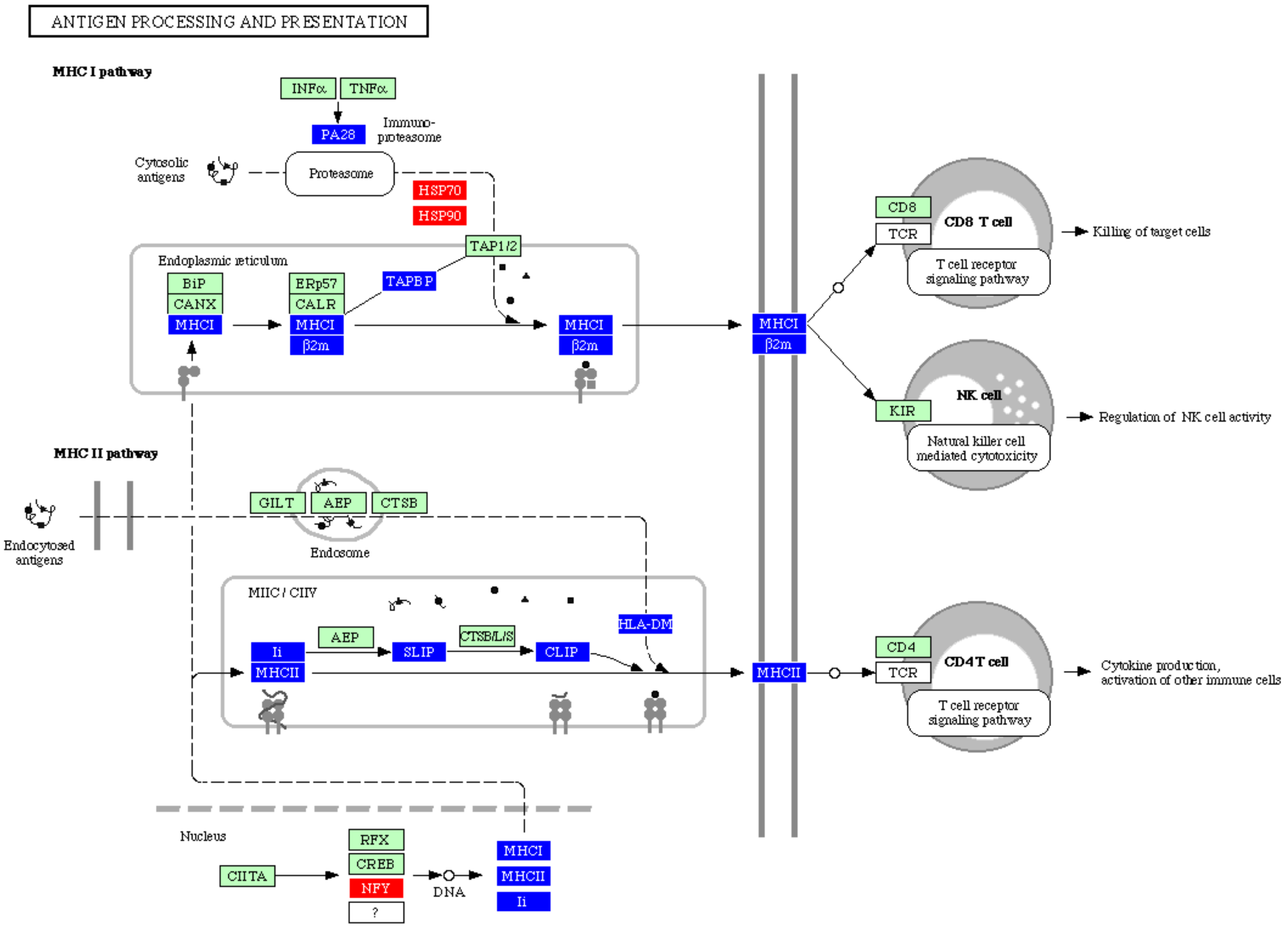

Additional file 4B.

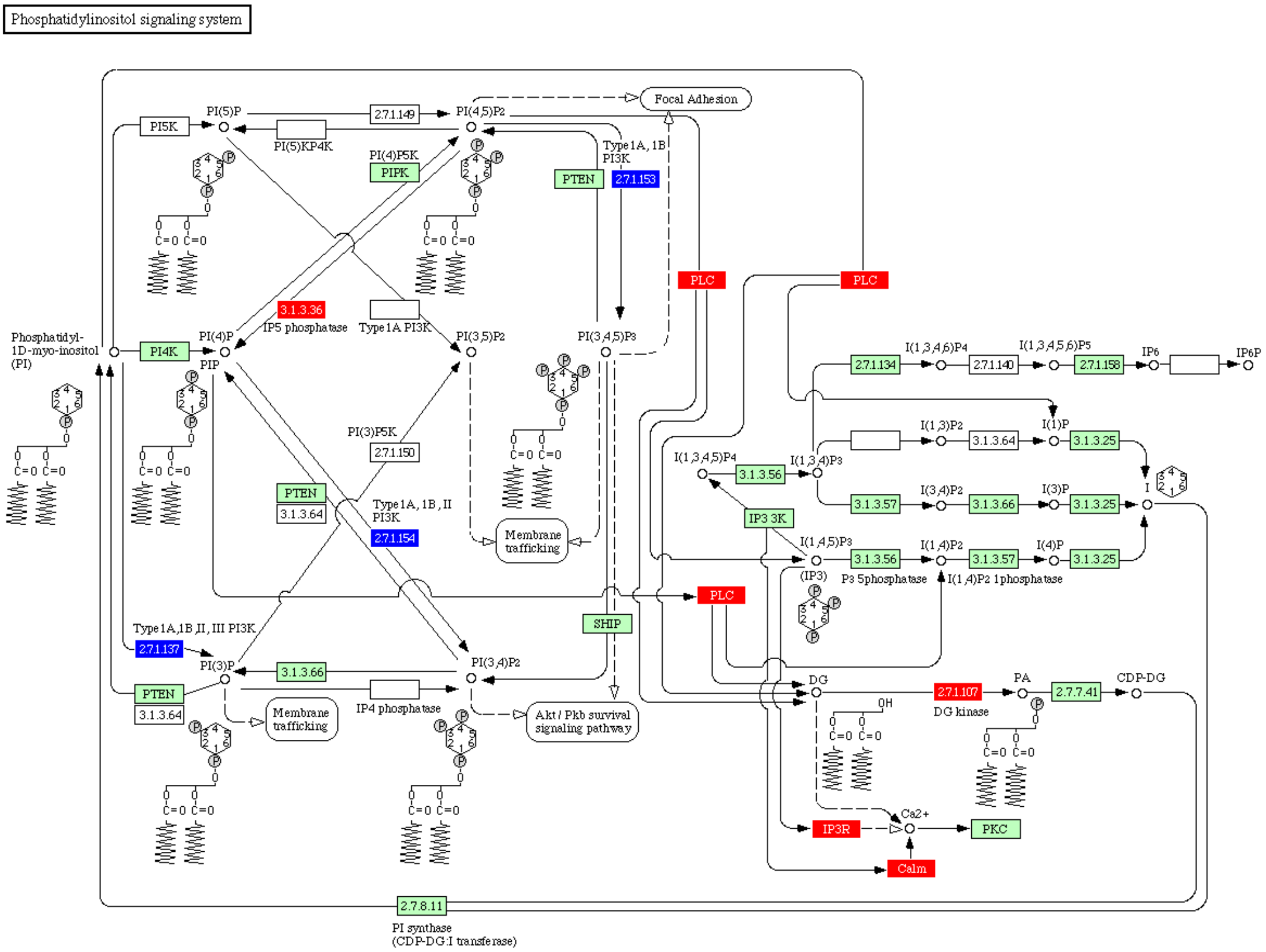

Additional file 4C.

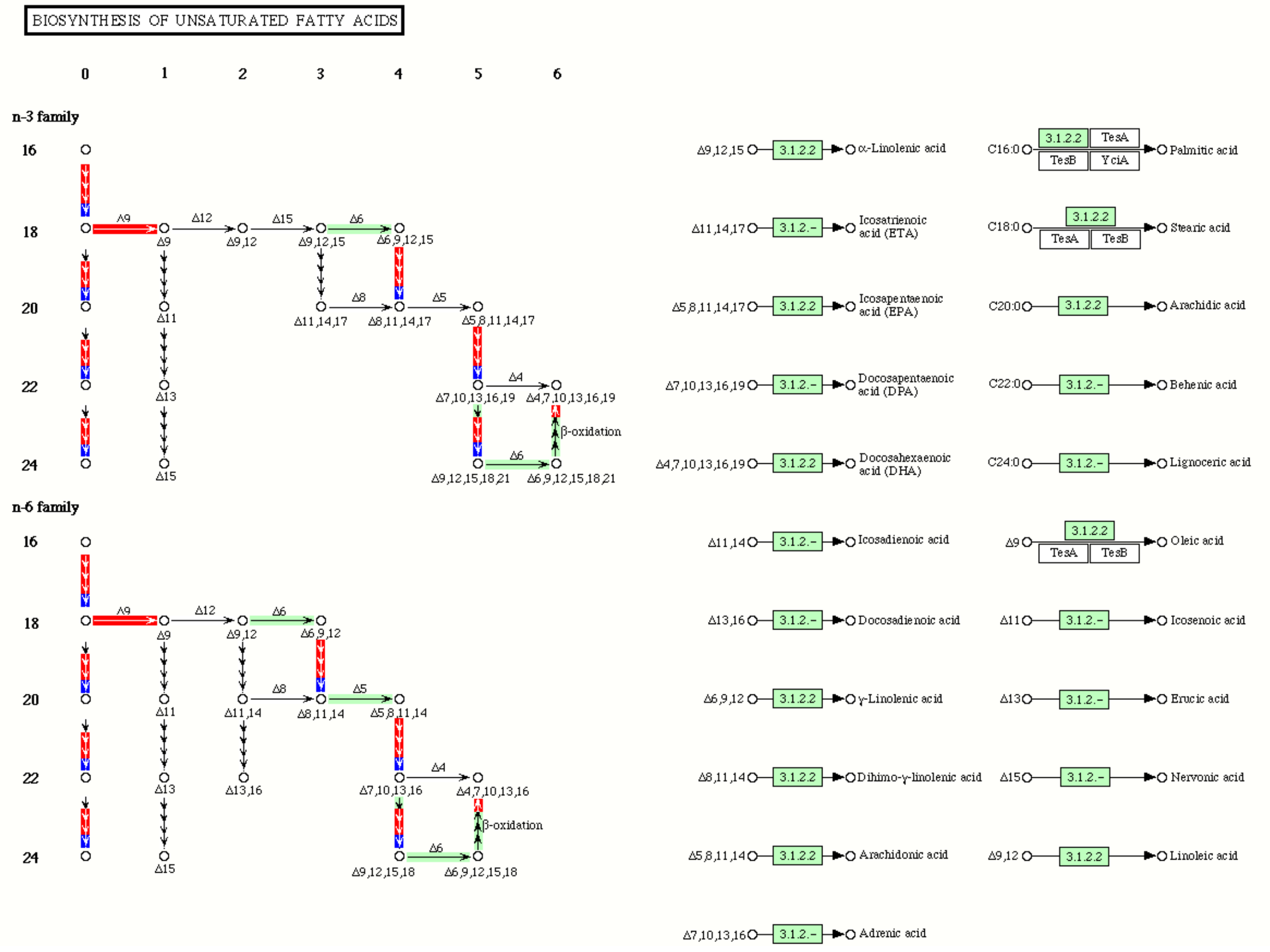

Additional file 4D.

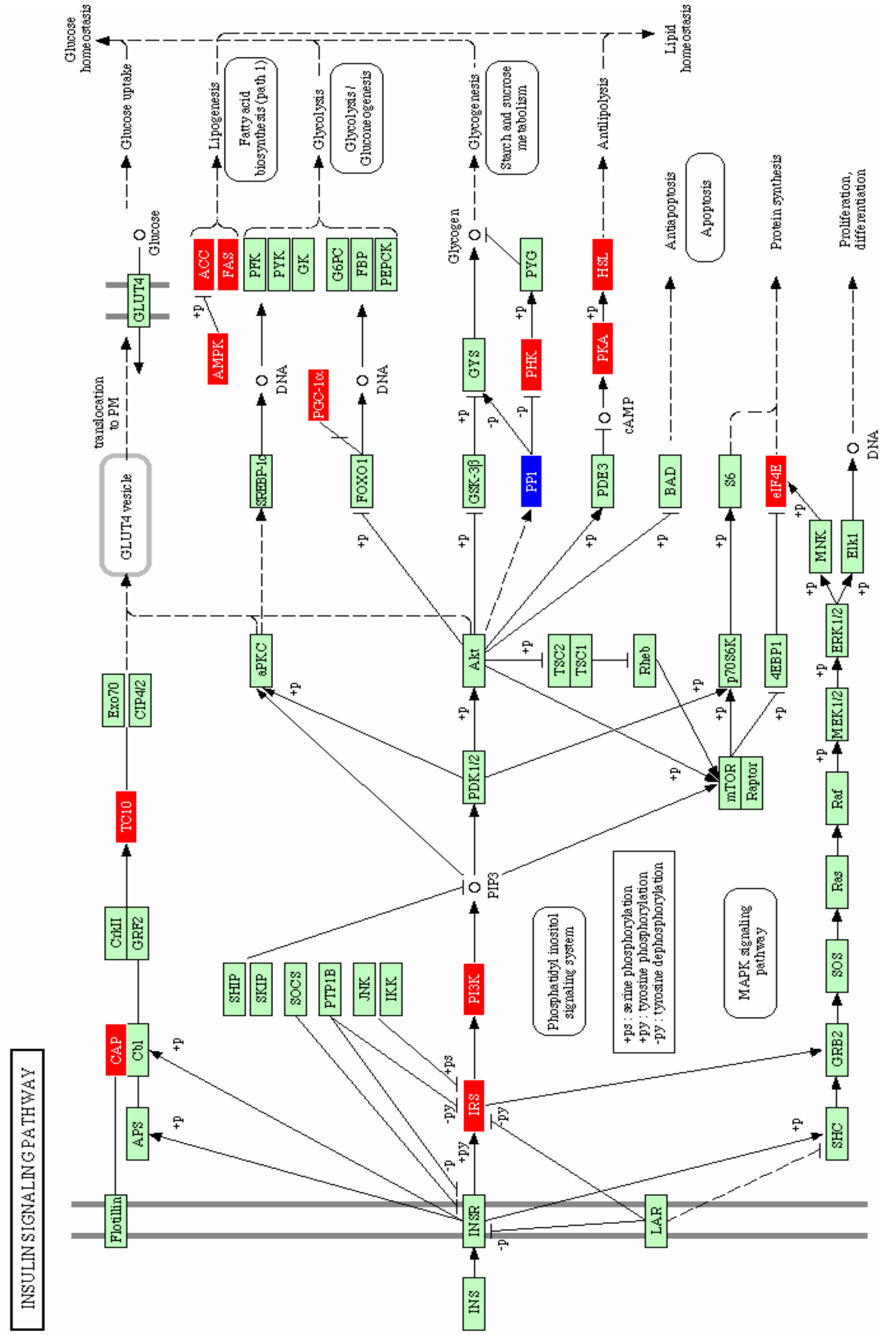

Additional file 4E.

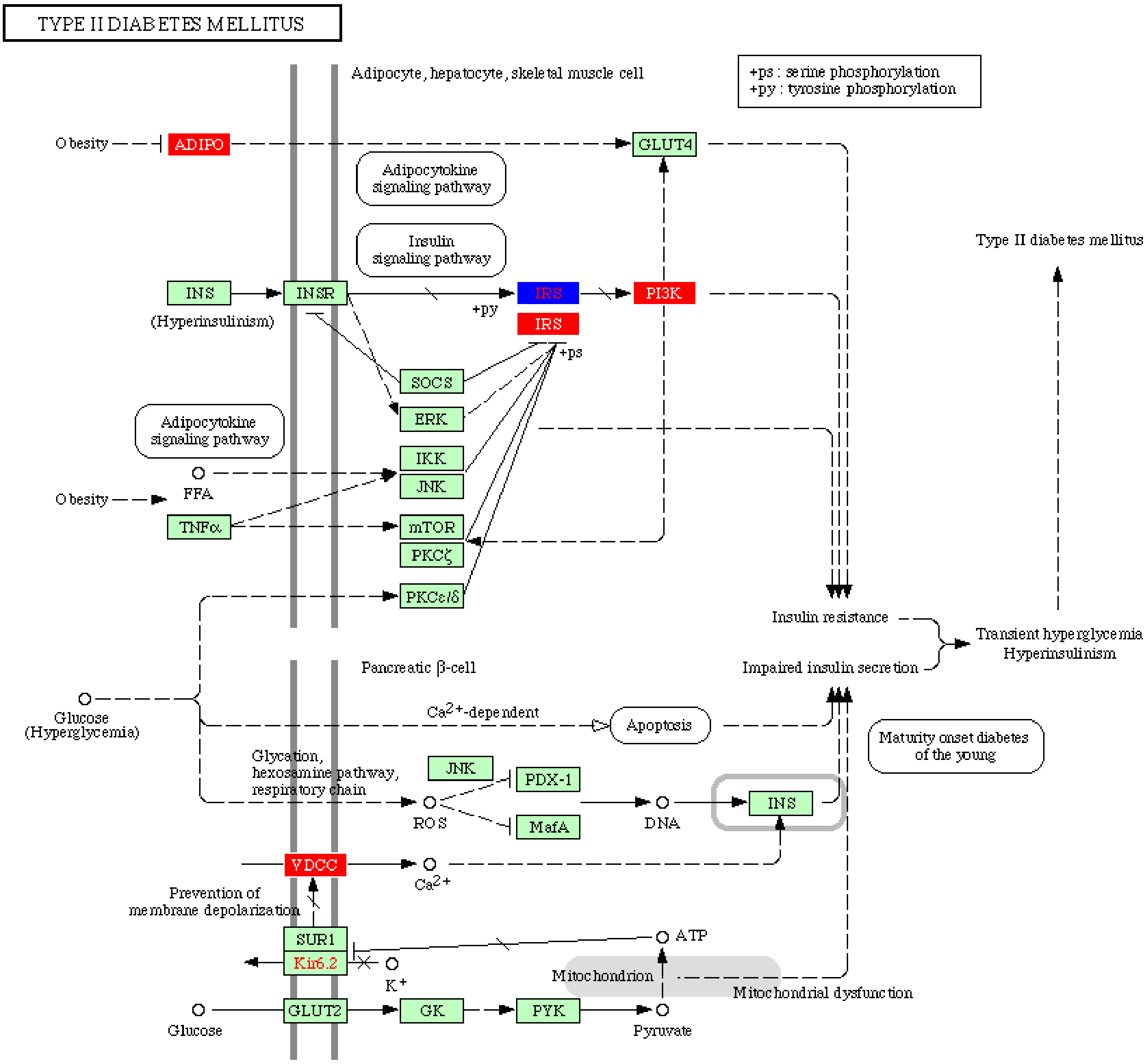

Additional file 4F.

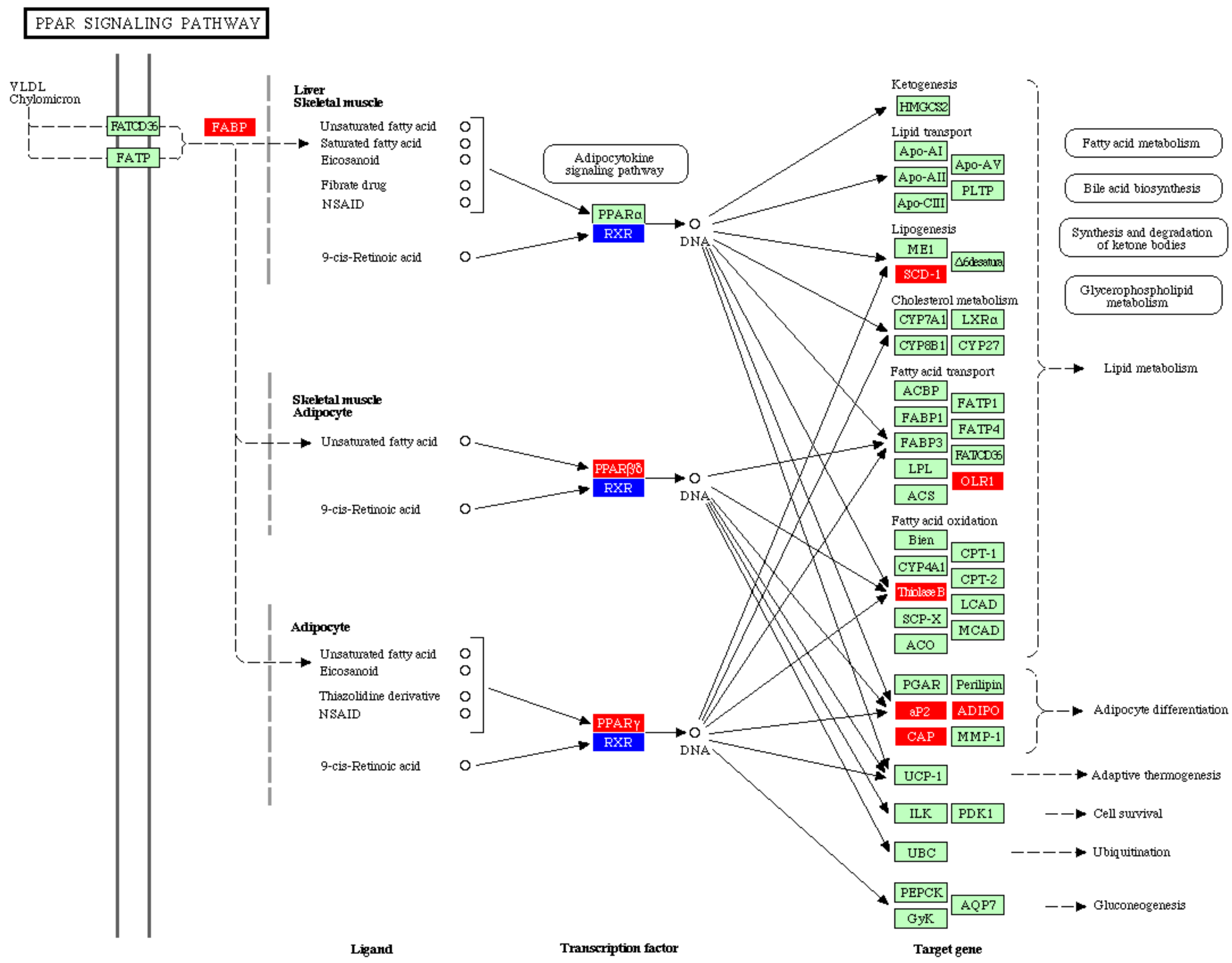

Additional file 4G.

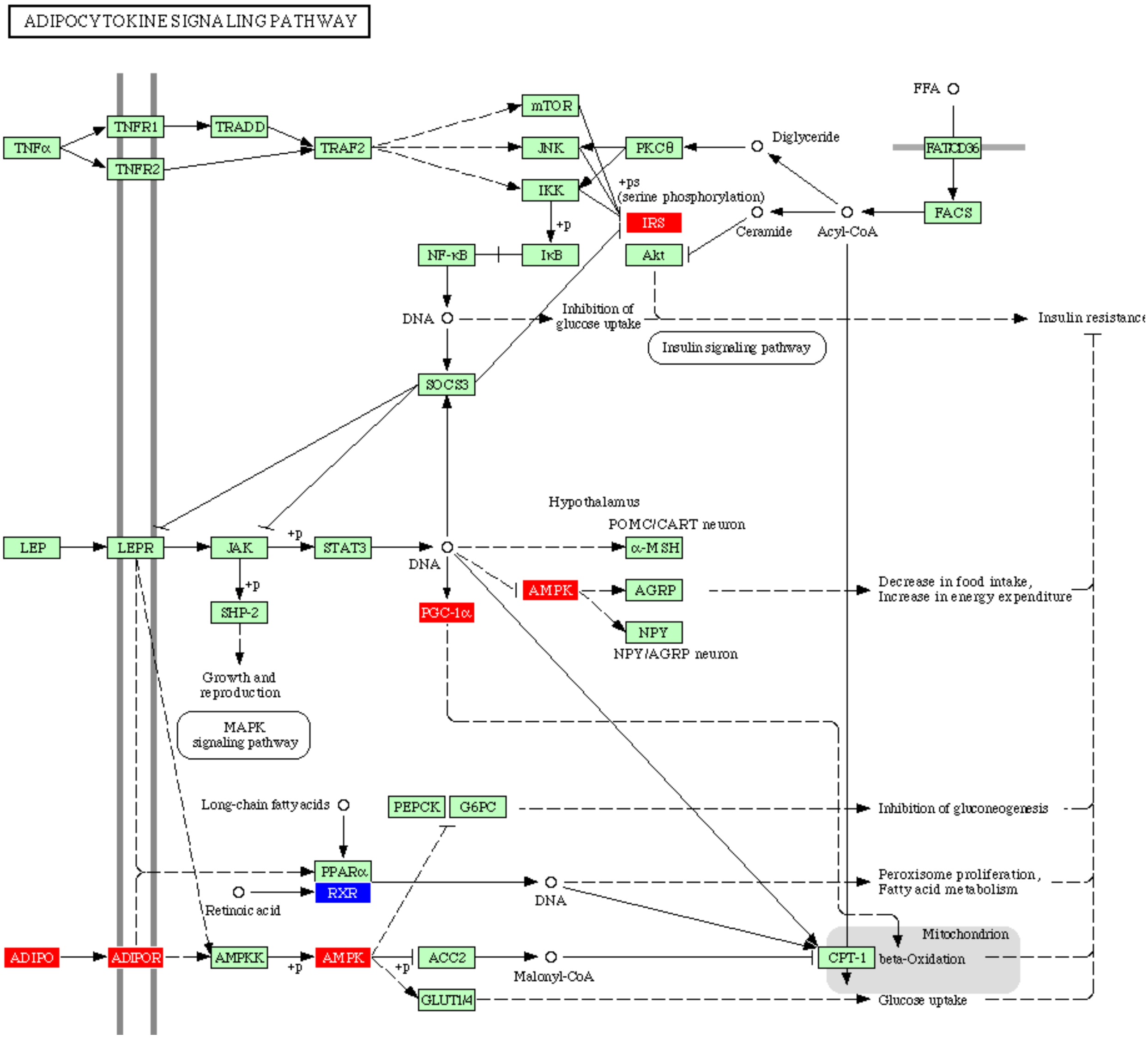

Additional file 4H.

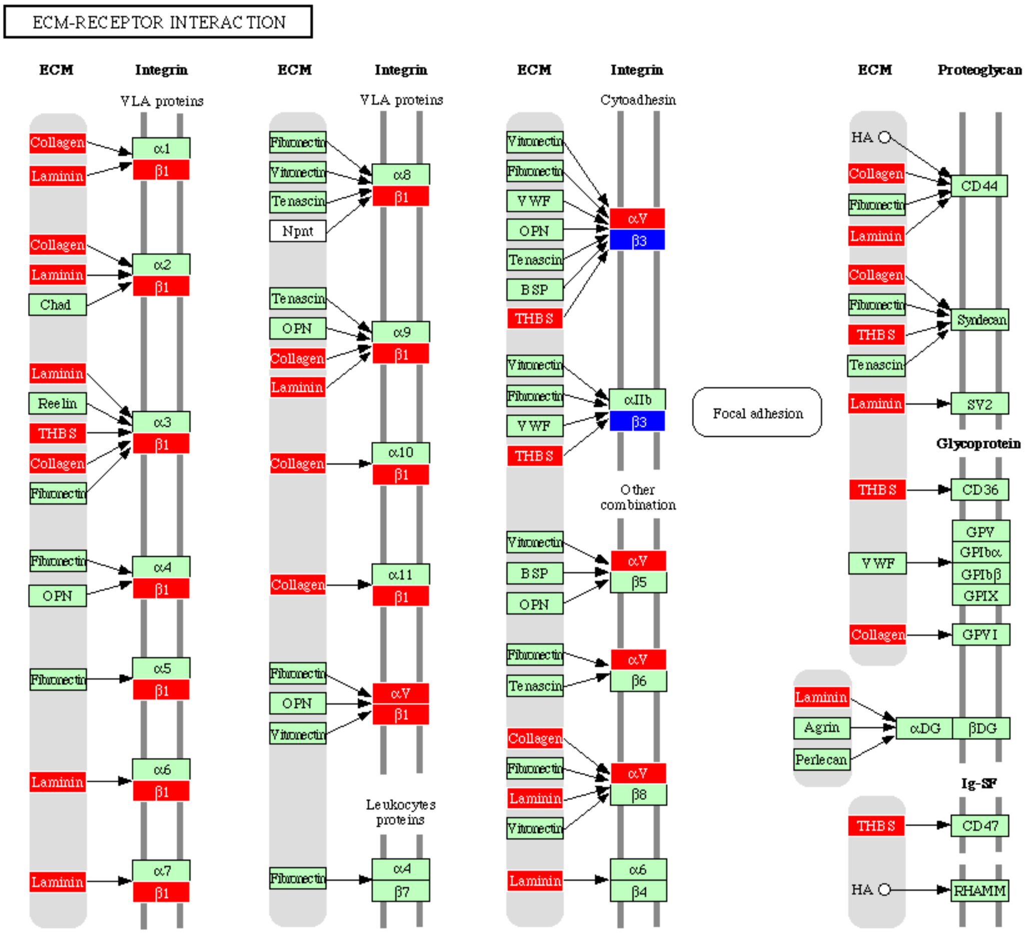

Additional file 4I.

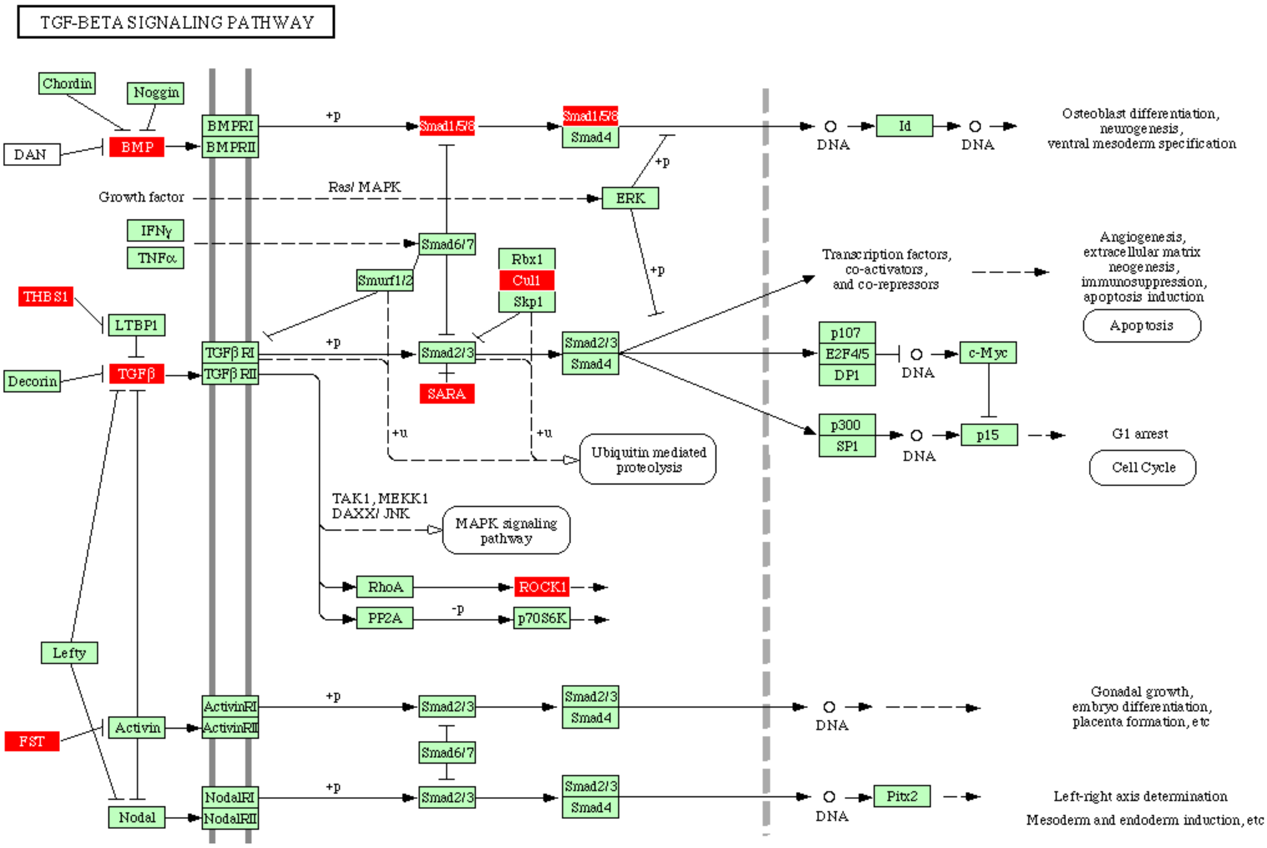

Supplement: Additional file 4 — Graphical representation of KEGG diagrams. Graphical representation of microarray results over the nine KEGG pathways significantly affected by the genes differentially expressed between the HIGH and LOW groups (p < 0.05). A: Antigen Processing and Presentation; B: Phosphatidylinositol Signalling; C: Biosynthesis of Unsaturated Fatty Acids; D: Insulin Signalling; E: Type II Diabetes Mellitus; F: PPAR Signalling; G: Adipocytokine Signalling; H: ECM-receptor Interaction; I: TGF-beta Signalling. In red, genes overexpressed in the HIGH group. In blue, genes overexpressed in the LOW group. [file 1471-2164-11-372-S4.PDF]
