# Supplementary material for: Muscle transcriptomic profiles in pigs with divergent phenotypes for fatness traits
Source: BMC Genomics. 2010 Jun 11;11:372. doi: 10.1186/1471-2164-11-372 (PMC2894043; doi:10.1186/1471-2164-11-372)
Supplement: Additional file 5 — List of genes selected for real time qPCR validation. Genes were selected for their involvement in lipid metabolism or muscle/fat development. A brief summary describing gene function and involved in KEGG pathways is provided. [file 1471-2164-11-372-S5.PDF]

Additional file 5: List of genes selected for real time qPCR validation. Genes were selected for their involvement in lipid metabolism or muscle / fat development. A summary of the gene function and involved in KEGG pathways is indicated.

Symbol	Gene Name	Function	KEGG Pathways (*H. sapiens*)
**ACACA**	Acetyl-CoA carboxylase 1	Biosynthesis of fatty acids	PATH: hsa00061 Fatty acid biosynthesis<br>PATH: hsa00620 Pyruvate metabolism<br>PATH: hsa00640 Propanoate metabolism<br>PATH: hsa04910 Insulin signaling pathway
**ADIPOR2**	Adiponectin receptor protein 2	Adiponectin receptor. Adipocyte differentiation.	PATH: hsa04920 Adipocytokine signaling pathway
**AGPAT2**	1-acyl-sn-glycerol-3-phosphate acyltransferase beta	Assiciated with fat tissue differentiation. Mutation of this gene causes lipodystrophies.	PATH: hsa00561 Glycerolipid metabolism<br>PATH: hsa00564 Glycerophospholipid metabolism<br>PATH: hsa00565 Ether lipid metabolism
**APOE**	Apolipoprotein E	Transport of plasma lipids	PATH: hsa01510 Neurodegenerative Diseases<br>PATH: hsa05010 Alzheimer's disease
**AQP4**	Aquaporin 4	Water channel expressed at very high levels in muscle. Function unknown.	
**CEBPD**	CCAAT/enhancer binding protein delta	Factor that starts adipose differentiation in pig	
**CES3**	Liver carboxylesterase 1 precursor (ACAT)	Cholesterol estherification	PATH: hsa00960 Alkaloid biosynthesis II<br>PATH: hsa00983 Drug metabolism - other enzymes
**CIDEA**	Cell death activator CIDE-A	Triglycerides storing in fat droplets.	
**DGAT2**	diacylglycerol O-acyltransferase homolog 2	Synthesis of triglycerides	PATH: hsa00561 Glycerolipid metabolism<br>PATH: hsa00830 Retinol metabolism
**ELOVL6**	long-chain fatty-acyl alongase family member 6	Elongation of long-chain fatty acids.	PATH: hsa01040 Biosynthesis of unsaturated fatty acids
**FABP4**	Fatty acid-binding protein, adipocyte	Uptake of lipids to the cell cytoplasm	PATH: hsa03320 PPAR signaling pathway
**FASN**	Fatty acid synthase	Fatty acids biosynthesis	PATH: hsa00061 Fatty acid biosynthesis<br>PATH: hsa01040 Biosynthesis of unsaturated fatty acids<br>PATH: hsa04910 Insulin signaling pathway
**IGFBP5**	Insulin-like growth factor binding protein 5 precursor	Regulation of IGFI/IGF2 growth factors	
**IRS2**	Insulin receptor substrate-2	Initiator of insulin signal transduction	PATH: hsa04910 Insulin signaling pathway<br>PATH: hsa04920 Adipocytokine signaling pathway<br>PATH: hsa04930 Type II diabetes mellitus

**LIPE**	Hormone sensitive lipase	Hydrolyse stored triglycerides to free fatty acids	PATH: hsa04910 Insulin signaling pathway
**LPIN1**	Lipin 1	Uptake of lipids to the cell cytoplasm	
**LRP11**	Low-density lipoprotein receptor-related protein 11 precursor.	Receptor activity	
**MEF2A**	Myocyte-specific enhancer factor 2A	Muscular differentiation factor. Inhibits adipose tissue differentiation	
**NPC1**	Niemann-Pick C1 protein precursor	Uptake of cholesterol to the cell cytoplasm	
**PIK3C2A**	phosphoinositide-3-kinase, class 2, alpha polypeptide	Secondary messenger	PATH: hsa04070 Phosphatidylinositol signaling system
**PIK3R1**	Phosphatidylinositol 3-kinase regulatory alpha subunit	Secondary messenger associated to diabetes mellitus. Regulates lipitin plasma levels.	PATH: hsa04012 ErbB signaling pathway<br>PATH: hsa04070 Phosphatidylinositol signaling system<br>PATH: hsa04150 mTOR signaling pathway<br>PATH: hsa04210 Apoptosis<br>PATH: hsa04370 VEGF signaling pathway<br>PATH: hsa04510 Focal adhesion<br>PATH: hsa04620 Toll-like receptor signaling pathway<br>PATH: hsa04630 Jak-STAT signaling pathway<br>PATH: hsa04650 Natural killer cell mediated cytotoxicity<br>PATH: hsa04660 T cell receptor signaling pathway<br>PATH: hsa04662 B cell receptor signaling pathway<br>PATH: hsa04664 Fc epsilon RI signaling pathway<br>PATH: hsa04670 Leukocyte transendothelial migration<br>PATH: hsa04810 Regulation of actin cytoskeleton<br>PATH: hsa04910 Insulin signaling pathway<br>PATH: hsa04930 Type II diabetes mellitus<br>PATH: hsa05210 Colorectal cancer<br>PATH: hsa05211 Renal cell carcinoma<br>PATH: hsa05212 Pancreatic cancer<br>PATH: hsa05213 Endometrial cancer<br>PATH: hsa05214 Glioma<br>PATH: hsa05215 Prostate cancer<br>PATH: hsa05218 Melanoma<br>PATH: hsa05220 Chronic myeloid leukemia<br>PATH: hsa05221 Acute myeloid leukemia

			PATH: hsa05222 Small cell lung cancer<br>PATH: hsa05223 Non-small cell lung cancer
**PPARD**	Peroxisome proliferator activated receptor delta	Activator of lipogenesis and adipose tissue differentiation	PATH: hsa03320 PPAR signaling pathway<br>PATH: hsa04310 Wnt signaling pathway<br>PATH: hsa05221 Acute myeloid leukemia
**PPARG**	Peroxisome proliferator activated receptor gamma	Activator of lipogenesis and adipose tissue differentiation	PATH: hsa03320 PPAR signaling pathway<br>PATH: hsa05216 Thyroid cancer
**PPARGC1A**	Peroxisome proliferator activated receptor gamma coactivator 1 alpha	Activator of lipogenesis and adipose tissue differentiation	PATH: hsa04910 Insulin signaling pathway<br>PATH: hsa04920 Adipocytokine signaling pathway
**RXR**	Retinoic acid receptor RXR-Gamma	Cholesterol homeostasis; Gene transactivation in response to bioactive lipid uptake in peripheral cells	PATH: hsa03320 PPAR signaling pathway<br>PATH: hsa04920 Adipocytokine signaling pathway<br>PATH: hsa05216 Thyroid cancer<br>PATH: hsa05222 Small cell lung cancer<br>PATH: hsa05223 Non-small cell lung cancer
**SCD**	Acyl-CoA desaturase	Fatty acid desaturation	PATH: hsa01040 Biosynthesis of unsaturated fatty acids<br>PATH: hsa03320 PPAR signaling pathway
**TBC1D1**	TBC1 domain family member 1	Regulation of the glucose transporter GLUT4 availability; Related to obesity syndrome	PATH: hsa04910 Insulin signaling pathway
**VEGFA**	Vascular endothelial growth factor A precursor	Vascular permeability, inducing angiogenesis and vasculogenesis	PATH: hsa04060 Cytokine-cytokine receptor interaction<br>PATH: hsa04150 mTOR signaling pathway<br>PATH: hsa04370 VEGF signaling pathway<br>PATH: hsa04510 Focal adhesion<br>PATH: hsa05211 Renal cell carcinoma<br>PATH: hsa05212 Pancreatic cancer<br>PATH: hsa05219 Bla dder cancer
CAV1	*Caveolin-1*	*Cholesterol binding*	*Excluded from analysis → wrong annotation*
LIPA	*Lysosomal acid lipase/cholesteryl ester hydrolase*	*Cholesterol and fatty acid estherification*	*Excluded from analysis → wrong annotation*
OSBPL6	*Oxysterol binding protein-related protein6*	*Intracellular lipid receptors. HDL-levels regulator*	*Excluded from analysis → wrong annotation*
